# Aerial imagery and Segment Anything Model for architectural trait phenotyping to support genetic analysis in peanut breeding

**DOI:** 10.1016/j.plaphe.2025.100126

**Published:** 2025-10-27

**Authors:** Javier Rodriguez-Sanchez, Raissa Martins Da Silva, Ye Chu, Lenin Rodriguez, Jing Zhang, Kyle Johnsen, Peggy Ozias-Akins, Changying Li

**Affiliations:** a School of Electrical and Computer Engineering, University of Georgia, Athens, GA, USA; b Department of Crop and Soil Sciences, University of Georgia, Tifton, GA, USA; c Department of Horticulture, University of Georgia, Tifton, GA, USA; d Department of Horticultural Science, North Carolina State University, Raleigh, NC, USA; e Department of Agricultural and Biological Engineering, University of Florida, Gainesville, FL, USA

**Keywords:** UAS phenotyping, SAM, Convolutional neural network, Peanut architecture, QTL mapping

## Abstract

Unmanned aerial systems (UAS) are reliable tools for field phenotyping, enabling rapid, large-scale, and cost-effective data collection to support breeding programs. However, many UAS-based approaches rely on manual data processing, limiting scalability and efficiency. This study presents a fully automated pipeline for high-throughput phenotyping (HTP) of peanut crop architectural traits, including canopy height (CH), growth habit (GH), and mainstem prominence (MP) by integrating UAS imagery, a vision foundation model—Segment Anything Model (SAM), and convolutional neural networks (CNN). SAM auto-mask generator mode was used to identify field extent and orientation, while SAM interactive mode enabled individual plot segmentation using auto-generated point prompts. Terrain points automatically sampled near each plot were used to model the ground surface and compute the canopy height model, allowing CH estimations at the plot level. CH estimations showed strong agreement with manual measurements (R² ​= ​0.78, RMSE ​= ​3 ​cm, MAPE ​= ​10 ​%). For MP and GH estimation, three pre-trained CNN models (AlexNet, ResNet18, and EfficientNet-B0) were evaluated, with AlexNet achieving the highest accuracy (89 ​% for GH, 83 ​% for MP). To assess the feasibility of using these HTP-derived estimations in plant breeding, quantitative trait loci (QTL) analysis was performed, identifying major-effect loci associated with these traits. The results were consistent with conventional QTL mapping methods, demonstrating that UAS-based phenotyping provides reliable trait data for genetic studies in peanut breeding. Overall, our deep learning-based data processing workflow minimizes manual efforts, providing an efficient and scalable approach that can accelerate genetic studies and trait selection in large-scale breeding programs.

## 1 Introduction

Peanut (*Arachis hypogaea* L.) production is an important component of U.S. agriculture, primarily grown for food processing and exports. In 2023, the U.S. planted 665,708 ​ha of peanuts, generating over $1.6 billion in production value, making it the seventh most important crop in the country [1]. As the fourth largest peanut producer, following China, India, and Nigeria, the U.S. accounts for approximately 5 ​% of global peanut production. To remain competitive and meet growing demands, sustained genetic advancements are essential for improving yield, quality, and resilience to biotic and abiotic stresses. Breeding programs play a crucial role in developing high-performing varieties that enhance productivity and adaptability, ensuring long-term sustainability and economic viability [2].

The integration of high-throughput phenotyping (HTP) technologies in breeding programs has become increasingly important for accelerating crop improvement. Accurate phenotyping of plant architectural traits is essential for peanut breeding, as these traits influence productivity, adaptability, and mechanical harvestability [3]. However, conventional phenotyping methods, based on manual measurements and visual assessments, are labor-intensive, prone to human error, and difficult to scale [4], especially in crops with complex branching patterns like peanut. HTP facilitates large-scale automated data collection, contributing to larger and more robust datasets for genomic selection or trait association studies, thereby enhancing breeding efficiency [5].

Among HTP methods, unmanned aerial systems (UAS) have become an effective tool for non-destructive, high-resolution data acquisition in large experimental fields. Equipped with RGB (red-green-blue), multispectral, or light detection and ranging (LiDAR) sensors, UAS platforms enable rapid assessment of diverse phenotypes under heterogeneous field conditions [6,7]. In peanut breeding, UAS-based phenotyping has been applied for assessing traits such as canopy height [8,9], maturity [10], and drought tolerance [11], as well as for early disease detection [12,13], leaf area index (LAI) monitoring [14], and chlorophyll content analysis [15]. However, the application of UAS for detailed architectural trait assessment, such as growth habit (GH) and mainstem prominence (MP), remains uncommon. Extracting these traits from UAS imagery requires efficient data processing workflows, which remain key technical challenges in deploying UAS-based phenotyping at scale [16].

Despite advances in UAS data acquisition, integrating UAS-based phenotyping into routine breeding programs is still hindered by challenges in image processing and trait extraction. Traditional aerial data processing often require manual input and technical expertise, making them time-consuming and difficult to scale. A key bottleneck is the segmentation of crop plots, which serves as the foundation for extracting spatially localized phenotypic information. Dense breeding fields, with closely spaced plants and heterogeneous growth, further complicate segmentation and have been the focus of extensive research [17]. Although manual methods have been increasingly replaced by computer vision algorithms and advanced software tools [18,19], field heterogeneity remains challenging [20]. As imaging datasets expand in scale and complexity, there is an increasing need for robust, scalable, and fully automated data processing pipelines to support HTP in crop breeding [21].

Accurate individual plot segmentation from UAS imagery remains a key challenge in HTP pipelines. Tools such as Easy MPE [18] and GRID [20] offer important advancements by enabling semi-automatic or supervised plot delineation. However, both exhibit critical limitations that hinder scalability and broader applicability. Easy MPE, for example, assumes uniform crop rows with minimal weed interference, restricting its use to early growth stages before canopy closure and inter-row overlap. It is also sensitive to light and color variation when using basic vegetation index-based segmentation, often requiring more complex, manually trained alternatives. GRID, while more sophisticated, still relies on assumptions of grid-like planting patterns, which may fail in irregular or non-uniform fields. Moreover, both tools often require user-defined parameters (e.g., number of clusters, channel selection in GRID) and manual refinement through graphical interfaces, limiting full automation and demanding technical expertise. These constraints highlight the need for robust, scalable, and fully automated data processing pipelines capable of generalizing across species and field conditions to effectively support HTP in crop breeding.

Building on the need for scalable plot-level data extraction, recent progress in machine learning (ML), deep learning (DL), and vision foundation models, particularly the Segment Anything Model (SAM) [22], have opened new opportunities to fully automate agricultural imagery without requiring annotated training data. SAM has shown strong zero-shot performance in generating accurate segmentation masks from remote sensing imagery [23], making it a promising tool for UAS-based phenotyping. However, its accuracy depends on image resolution and scene complexity, whereas cluttered agricultural environments may lead to over-segmentation [24]. Domain-specific adaptations [25] and prompt optimization [26] are necessary to tailor SAM to agricultural contexts. Leveraging SAM while addressing these challenges could significantly reduce human input and improve scalability in UAS-based phenotyping workflows, facilitating broader adoption in breeding programs.

Beyond image segmentation, ML and DL methods provide robust frameworks for automating trait estimation from remote sensing. In peanut crops, ML-based UAS phenotyping has primarily focused on yield estimation [27,28], but has also been applied to traits such as LAI and leaf greenness, providing insights into plant health and vigor [29]. DL approaches have further enabled biomass prediction and pod count estimation [30]. Although advancements have been made, the widespread deployment of HTP in peanut breeding is limited by bottlenecks in data processing and trait extraction, which compromise consistency and reduce reproducibility. Automating key steps in data processing can minimize manual intervention, improve reproducibility, and enhance the scalability of UAS-based phenotyping in breeding programs.

Despite the increasing use of HTP traits derived from remote sensing data in QTL mapping and genome-wide association studies (GWAS) for major crops, its application in peanut breeding remains underexplored. While HTP has been used to identify loci associated with yield components, physiological traits, and stress responses in other crops, its use in genetic analysis for architectural traits in peanuts is limited, presenting a missed opportunity for advancing breeding efforts. Traits such as GH and MP have not been well characterized using HTP data, and their integration into genetic mapping remains largely unexplored. This is partly due to the difficulty of measuring these traits manually in the field, especially on a large scale. However, both GH and MP are important for determining canopy structure and light interception, and have been associated with yield potential and adaptability in peanut breeding programs [31]. This study aims to address this gap by developing and evaluating ML-based approaches for the HTP of these key architectural traits in peanut breeding using UAS data, enhancing the efficiency of genetic studies.

The main goal of this study was to develop and evaluate ML-based methods for HTP of architectural traits in peanut breeding using UAS data. The specific objectives were to: (1) develop a fully automated workflow for processing aerial orthomosaic images using the SAM vision foundation model; (2) automate canopy height (CH) estimation from UAS-derived digital surface models and assess its accuracy against ground truth measurements; (3) train and evaluate different CNN models for estimating growth habit (GH) and mainstem prominence (MP); and (4) integrate HTP-derived MP and GH estimations into QTL analysis to assess their effectiveness relative to traditional manual phenotyping methods.

The primary contributions of this study are centered on addressing critical limitations in HTP data processing:

1. Zero-shot, fully automated plot segmentation: We introduce a novel pipeline that uses the Segment Anything Model (SAM) to segment plots from UAS imagery without annotated training data or manual parameter tuning, overcoming key limitations of existing tools.
2. Adaptation to complex field heterogeneity: Leveraging SAM's zero-shot capabilities, our pipeline effectively segments plots in heterogeneous environments, handling inter-row overlap, misaligned planting, and irregular plant growth, without the need for crop-specific training, making it broadly applicable across species and planting designs.
3. Integration of local terrain sampling for CH estimation: Our method incorporates local DTM sampling adjacent to each plot mask, improving CH estimation accuracy by minimizing the impact of terrain artifacts such as wheel furrows or pivot tracks. This plot-specific terrain correction contrasts with global DTMs used in many prior pipelines.
4. Scalable phenotyping pipeline: Beyond plot segmentation, we extend our framework to estimate key agronomic traits such as GH and MP via CNN, providing a holistic phenotyping solution integrated with plot segmentation and height estimation.

Together, these contributions shift plot segmentation from parameter-dependent, rule-based methods to a foundation model–driven approach, advancing HTP toward greater autonomy and scalability.

## 2 Materials and methods

### 2.1 Experimental field and ground truth measurements

**Fig. 1:**
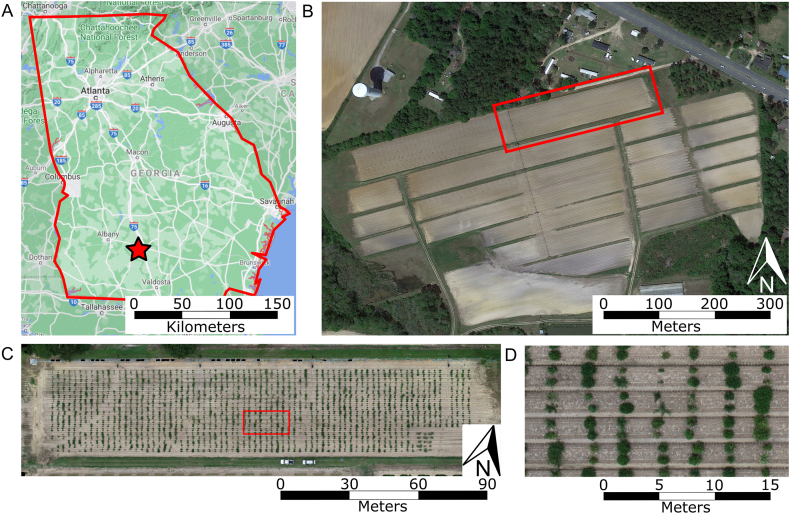
Experimental field location and layout. The experiments were conducted at the Gibbs Farm research station, Tifton, GA, US. (A) Map of Georgia showing the experimental location (red star). (B) Aerial image of Gibbs Farm with the experimental field outlined in red. (C) Close-up aerial view of the experimental field. (D) Zoomed view of the plot arrangement within the area highlighted in panel (C).

This study was conducted at the University of Georgia research station in Gibbs Farm, Tifton, GA (Fig. 1). Plant material was planted in early June 2022. Three peanut populations—GT, C1803, and IF—were hand-planted in a two-row plot arrangement, on raised beds that extended the full length of the field and measured 1.82 ​m in width. Each plot consisted of two rows spaced 0.91 ​m apart, with three seeds per row planted at 15-cm intervals, resulting in a planted length of approximately 0.5 ​m. Plots were arranged along each raised bed, with 2-m alleys of bare soil separating adjacent plots in the row direction. A total of 1350 plots, representing approximately 450 unique breeding lines, were arranged in a randomized complete block design with three field replicates per line. The total field dimensions were 184 ​m ​× ​38 ​m (length ​× ​width).

The morphological traits analyzed in this study included canopy height (CH), growth habit (GH), and mainstem prominence (MP). Trait measurements were collected manually 90 days after planting (DAP), which corresponds to the reproductive stages R6 to R7 (full seed to beginning maturity) in most peanut cultivars [32]. At this stage peanut plants have typically completed their vegetative growth and reached maximum canopy size, making it an appropriate time point for evaluating plant height, canopy structure, and vigor. CH was measured as the distance from the ground to the top of the canopy using a measuring tape. These measurements were taken from 50 randomly selected plots, with two measurements per plot averaged to obtain the final CH value.

MP and GH were visually rated following standard peanut trait categorization [33]. GH was rated on a scale from 1 to 6 (1: prostrate, 2: spreading, 3: spreading and bunch, 4: bunch, 5: erect, 6: mixed). Classes 1, 2, 4, and 5 are described in Table S1, based on descriptions and diagrams from previous studies [33,34]. Classes 3 and 6 represent intermediate or mixed canopy architectures combining traits from the other types, without a clear dominant form. MP was scored on a scale from 1 to 4 based on the visibility of the mainstem (1: not apparent, mainstem is not visible and the plant appears bushy with branches from the base; 2: somewhat apparent, mainstem partially visible but partly covered by branches; 3: apparent, mainstem clearly visible and prominent; 4: mixed, unclear or variable mainstem structure).

### 2.2 Aerial data collection and pre-processing

Aerial imagery was collected using a Phantom 4 Pro V2 quadcopter (Shenzhen DJI Sciences and Technologies Ltd., Shenzhen, China) with an integrated red-green-blue (RGB) camera. The UAS was flown at an altitude of 30 ​m above ground level with an 80 ​% front and side overlap between images to ensure comprehensive coverage. The flight was conducted within 2 ​h of solar noon under clear weather conditions, following pre-programmed flight paths created using DroneDeploy software (DroneDeploy, Inc., California, US). Four 16 in ​× ​16 in ground control points (GCPs) were deployed at the corners of the field to aid in visual alignment and provide reference markers during image processing. Although these GCPs were not surveyed with GNSS or RTK-GPS equipment, they served as internal references for assessing the spatial accuracy and scale consistency of the image alignment and orthomosaic generation.

Collected RGB images were processed using Agisoft Metashape Professional 1.5.5 (Agisoft LLC, Russia) to generate orthorectified images, following the methodology described in Ref. [35]. This process involved estimating camera positions using generic pair preselection with the highest accuracy for photo alignment. The key point limit parameter was set to 120,000, while the tie point limit was adjusted to 10,000. GCPs were manually marked in multiple overlapping images within Agisoft Metashape. Scale bars were defined along the edges of each GCP using their known dimensions. Two scale bars per GCP were designated as control references for optimization, and the remaining two were used as check bars to independently validate the results. A dense point cloud was then generated using these estimated positions, with the ultra-high-quality setting and mild depth filtering. From this dense cloud, a digital surface model (DSM) was produced. Finally, an orthomosaic was created by stitching the orthorectified images together using mosaic blending mode.

The final project had a mean reprojection error of 0.38 pixels, with a ground sampling distance (GSD) of 0.93 cm/pixel. The mean error for the control scale bars was 0.35 ​cm, while the mean error for the check scale bars was 0.33 ​cm, indicating consistent scaling across the reconstruction. While the lack of georeferenced coordinates limited the use of the orthomosaic and DSM for absolute geolocation, this internal scaling approach ensured high relative accuracy across the field, suitable for trait extraction at the plot level.

### 2.3 Overview of data processing workflow

**Fig. 2:**
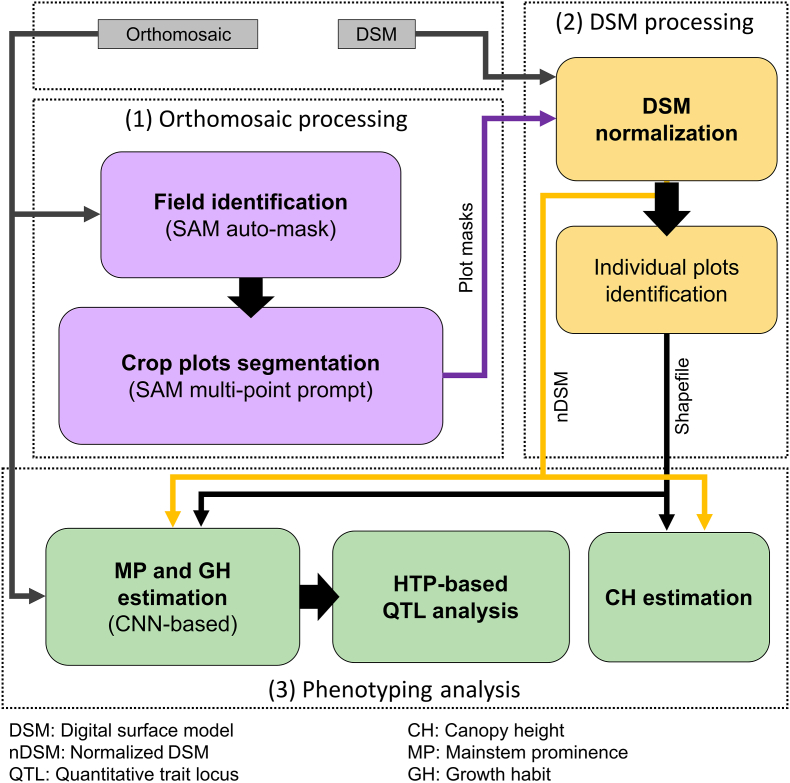
Schematic representation of the automated phenotyping pipeline, highlighting the main methodological contributions of this study. Arrows indicate the flow of data between processes. Colored boxes indicate the main contributions of this study.

The data processing workflow in this study aimed to automate key steps in UAS-based field phenotyping. Following aerial data collection and pre-processing—including orthomosaic and DSM generation—the pipeline was structured into three main steps (Fig. 2): (1) automated orthomosaic processing using the Segment Anything Model (SAM), (2) DSM processing and generation of plot shapefiles, which are a common geographic information system (GIS) vector format used to represent spatial features such as plot boundaries, and (3) high-throughput phenotyping (HTP) analysis.

The data processing pipeline was implemented in Python using PyTorch as the machine learning (ML) framework. All experiments were conducted on a desktop computer equipped with an 8-core Intel Core i7-9700K CPU (3.60 ​GHz) and 64 ​GB of RAM. To accelerate CNN model training and inference, an NVIDIA GeForce GTX 1080 (8 ​GB) GPU was used.

### 2.4 Automated orthomosaic processing

Following orthomosaic map generation, the Segment Anything Model (SAM) [22] was used to automate field plot segmentation, including field boundary detection and individual plot mask extraction. Unlike traditional semantic segmentation networks that require labeled training data and retraining for each new dataset, SAM enables zero-shot segmentation via prompt-based queries, making it well-suited for automated plot extraction across diverse field conditions without manual annotation. SAM offers two operational modes: an automatic mode that generates segmentation masks for all objects in an image and an interactive mode that refines segmentation through user-provided foreground and background points. To achieve full process automation, both modes were applied sequentially.

Among the available vision transformer (ViT) backbones for SAM, we selected the pre-trained SAM model based on the largest backbone (ViT-H (“Huge”)) due to its higher capacity to capture fine-grained spatial details and complex boundary structures, as demonstrated by its superior segmentation performance compared to smaller ViT variants [22]. Larger pre-trained ViT-based models tend to outperform smaller transformers versions [36]. SAM ViT-H encoder was trained on a large and diverse dataset of 1.1 billion masks and was used in the original SAM paper as the reference architecture. Although we tested all three SAM backbones (ViT-B (Base), ViT-L (Large), and ViT-H), we found that ViT-H produced slightly more detailed masks, especially at the edges of crop plots, with only a marginal increase in computational load. This might appear counterintuitive, but due to the moderate resolution of orthomosaic-derived imagery, the resource demands of ViT-H remained manageable.

#### 2.4.1 SAM-based field identification

**Fig. 3:**
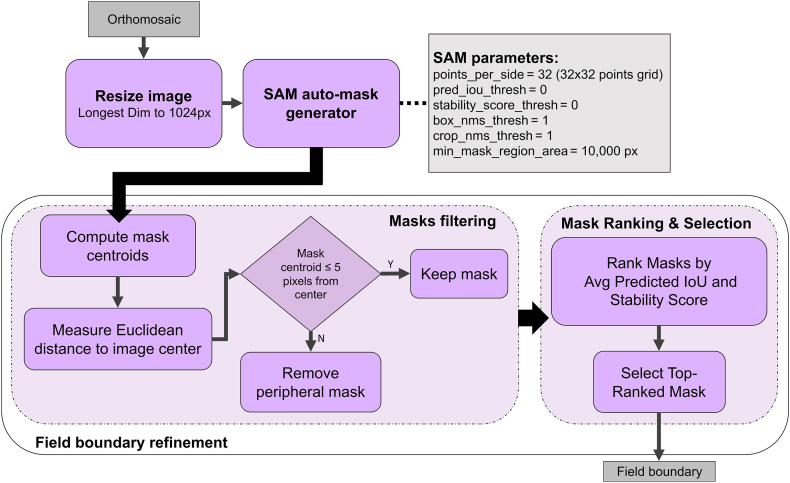
Overview of the automatic field boundary identification workflow using SAM. The process includes orthomosaic resizing, SAM-based segmentation with custom parameters, spatial filtering of candidate masks, and final selection based on predicted quality scores.

The first step in the UAS-based phenotyping workflow focused on automatically identifying the field boundary without manual annotation (Fig. 3). First, SAM automatic mode was applied to segment the orthomosaic using a dense grid of query points. A 32 ​× ​32 grid was used to ensure comprehensive coverage while maintaining an effective balance between segmentation details and processing efficiency. The longest image dimension was resized to 1024 pixels to optimize processing speed, following the original SAM implementation.

Several SAM segmentation parameters were adjusted to improve the completeness of retrieved masks (Table S2). These settings differ from SAM's default thresholds to better accommodate the lower resolution and overlapping features typical of aerial orthomosaic imagery. SAM generates up to three candidate masks per point, typically ranging from more specific to more generic interpretations of the target region. By default, SAM's automatic mask generator filters these candidates using quality thresholds—an Intersection-over-Union (IoU) threshold of 0.88 and a stability score threshold of 0.95. While appropriate for high-resolution, object-centric images, these thresholds are too restrictive for aerial field imagery, which lacks the fine-grained object detail required for reliable automatic filtering.

Additionally, SAM applies non-maximum suppression (NMS) by default (with a threshold of 0.7) to remove overlapping masks. However, in aerial imagery, this step can mistakenly eliminate useful masks due to natural overlap among adjacent plots and field structures. To prevent this, we disabled these filters during automatic segmentation. Both the IoU and stability score thresholds were set to zero to retain all candidate masks, and the NMS threshold was increased to 1 to preserve overlapping masks such as the full field boundary. This strategy enabled more flexible mask selection in the downstream post-processing pipeline.

After the initial segmentation, a post-processing procedure was applied to automatically refine the field boundary selection. SAM masks were first filtered based on their spatial location within the orthomosaic to exclude irrelevant segments, such as pathways, peripheral vegetation, and adjacent fields. To facilitate automatic field segmentation, we assumed that the experimental field was centrally located within the orthomosaic, consistent with standard UAS flight planning. The centroid of each mask was calculated as the center of its bounding box, and its Euclidean distance from the center of the orthomosaic was computed. Masks located near the orthomosaic center were prioritized, while peripheral masks, located farther than 5 pixels from the image center, were excluded to ensure that only the most relevant masks were retained.

The remaining masks were then ranked based on the predicted IoU and stability score metrics provided by SAM. The predicted IoU reflects confidence in mask accuracy, while the stability score quantifies segmentation consistency under varying conditions. By averaging these two metrics, the most stable and representative mask was selected as the final field boundary, ensuring precise delineation without manual input.

#### 2.4.2 SAM-based crop plot segmentation

**Fig. 4:**
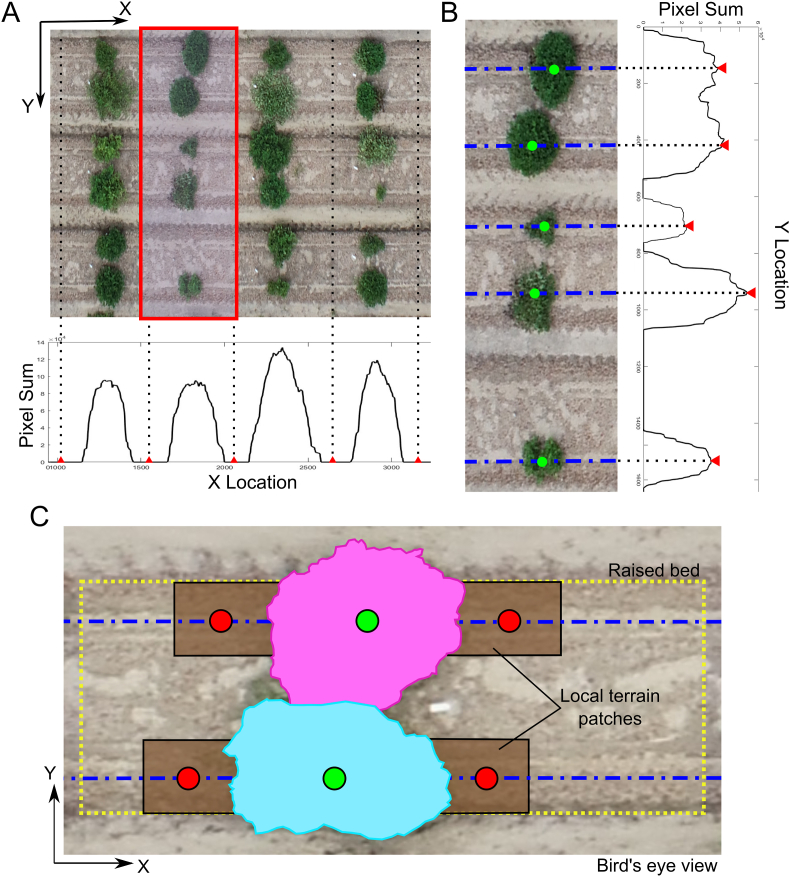
SAM-based plot segmentation and terrain sampling. (A) Crop ranges detection based on vertical summation of vegetation pixels. Red triangles mark the midpoints of regions with minimal vegetation, corresponding to inter-row alleys. (B) Zoomed-in view of the area highlighted by the red rectangle in panel (A). Red triangles indicate peaks in accumulated vegetation, corresponding to crop rows. (C) Distribution of sampling points used for local terrain estimation. SAM-generated masks for individual rows are shown in pink and turquoise. Blue dash-dotted lines represent the detected crop rows. Green circles denote plot centroids used as prompts for SAM, and red circles indicate terrain sampling points.

Once the field boundaries were identified, segmentation masks for individual crop plots were obtained using SAM interactive mode, which enables refined segmentation based on user-defined points. To fully automate this process, computer vision techniques were applied for point prompt selection (Fig. 4).

Vegetation pixels were first identified using the Excess Green minus Excess Red Index (ExG-ExR). Summing vegetation pixels along the vertical axis produced a distribution pattern where valleys indicated areas with minimal vegetation. The midpoints of these valleys defined the X-coordinates separating adjacent crop ranges (Fig. 4A). Each range was then processed individually (Fig. 4B). Summing vegetation pixels along the horizontal axis allowed the detection of the densest vegetation regions, which corresponded to plant rows (blue dash-dotted lines in Fig. 4B). The central points along these rows were designated as temporary plot centroids.

The detected centroids were used as multi-point input prompts in SAM interactive mode to generate segmentation masks. For each plot, its centroid served as a foreground point, while the centroids of neighboring plots were used as background points. The resulting segmentation masks were assigned unique plot IDs and served as the primary input for subsequent data processing and trait estimation.

### 2.5 Digital surface model processing

The digital surface model (DSM) captures elevation data essential for crop height estimation in UAS-based phenotyping. However, because the DSM represents a combination of ground elevation and plant canopy height, accurately measuring crop height requires isolating vegetation height from the underlying terrain. To achieve this, a normalized DSM (nDSM) was computed by subtracting the terrain elevation from the DSM, ensuring that only the vegetation height was retained for analysis. This process was necessary to account for variations in field topography that could otherwise introduce errors in plant height estimation.

Terrain elevation was estimated using a digital terrain model (DTM), constructed by sampling terrain points adjacent to each SAM-derived plot segmentation mask (Fig. 4C). Two sampling points were selected per plot, positioned on opposite sides of the segmentation mask, to represent ground elevation local to each plot. These points were then interpolated across the field using the *‘startinpy’* Python library, which implements a Delaunay triangulation algorithm to generate a continuous terrain surface. The nDSM was then computed by subtracting the DTM from the DSM.

Although four GCPs were deployed at the corners of the field as visual references, they were not surveyed with GNSS or RTK equipment. As a result, georeferencing relied solely on the onboard GNSS data embedded in image metadata, and outputs remained in a local coordinate system. Despite this limitation, the nDSM provided reliable relative canopy height estimates across the field, as the DSM and DTM were generated from the same image set and remained internally consistent. This approach effectively corrected for topographic variation and allowed robust comparison of canopy heights among plots.

#### 2.5.1 Individual plot identification and shapefile creation

Following nDSM computation, individual two-row plots were identified to generate shapefiles for extracting the geospatial data associated with each plot. To ensure a fully automated approach for defining plot boundaries, our methodology accounted for cases where some plots contained only one row due to poor germination or plant loss caused by disease. Since the crop was planted on raised beds, the DTM reconstruction represented the surface level of these beds, while tractor tracks appeared as lower elevation areas in the nDSM. These low-elevation regions served as a reference to identify the boundaries between adjacent planting beds.

To delineate plot boundaries, the horizontal lines separating each raised bed were detected by applying the Hough transform to the negative elevation values in the nDSM. These lines, combined with the previously identified crop range limits, defined the boundaries of each two-row plot. The final center for each plot was determined by computing the center of mass for the SAM-derived segmentation masks located between the plot boundaries. A bounding box of 224 ​× ​224 pixels, centered on the computed plot center, was then automatically drawn around the crop masks within each plot. The generated plot boundaries and associated metadata were stored in a shapefile (.shp) format for further data extraction.

### 2.6 Agronomic traits estimation

Following plot delineation, CH estimations were performed from the nDSM to quantify plant growth at the plot level. To obtain CH values, nDSM pixel values within each plot's bounding box from the shapefile were extracted and converted into 2D NumPy arrays of size 224 ​× ​224. This transformation ensured a standardized data structure for computational analysis. Since each plot contained two crop rows, the nDSM array was divided horizontally at the computed plot center, and the maximum pixel value (CH_max_) was extracted independently for each row. The final CH value for the plot was then computed as the average of the two row-level CH_max_ values. We also evaluated alternative metrics, including the 95th, 97th, and 99th percentiles, but the maximum value consistently yielded the best agreement with manually measured plant heights, achieving the highest R^2^ and lowest error metrics. This approach is particularly appropriate when plants are at or near full vegetative growth, and the tallest points in the canopy are biologically meaningful indicators of plant vigor.

After CH extraction from the nDSM, this structural information was leveraged alongside RGB imagery to estimate MP and GH using CNN models for image classification trained in the PyTorch framework. The plot's bounding boxes were used to extract individual plot images from both the orthomosaic and the nDSM. These images were used as input for the CNN-based classification model. While CH is also a relevant architectural trait, adequate data sample size is essential for model generalization and stable performance. Typically, several hundred accurately labeled samples, representing the full range of expected heights and diverse environmental conditions, are recommended, even when leveraging transfer learning [37]. Our dataset size of *N* ​= ​50 for CH ground-truth measurements was therefore considered insufficient for training a robust CNN regression model capable of generalizing across the observed variability in peanut canopy height.

To assess CNN classification performance, a comparative analysis was conducted across different models and data modalities. Three widely used CNN models were tested: AlexNet, ResNet, and EfficientNet, chosen for their balance between model complexity and computational efficiency. The selection of these CNN models was guided by three main factors: computational efficiency, prior benchmarking in agricultural phenotyping studies, and coverage of a range of network complexities. These models have been widely adopted in related work for trait classification and crop disease detection due to their robust performance and ease of implementation [[38], [39], [40]]. Our preliminary tests with varying backbone sizes indicated that deeper networks were prone to overfitting, likely due to the relatively low complexity of the classification task. Therefore, smaller models with fewer trainable parameters were preferred to enhance generalization—ResNet18 for ResNet and EfficientNet-B0 for EfficientNet. All models were initialized with pretrained weights and fine-tuned by training only the final fully connected layers.

Since MP and GH are inherently linked to plant structure, the inclusion of height information in the classification process was expected to improve predictive performance. To assess the impact of structural information on trait estimation accuracy, we evaluated each CNN model using three data modalities: RGB imagery alone, nDSM data alone, and a combined modality integrating RGB image channels with nDSM pixel data. In the combined modality, nDSM pixel values (originally in meters) were scaled by 100 to convert them into centimeters before being added to the G channel. Pixel values exceeding 255 were clipped to maintain consistency. This transformation embedded height information into the RGB image while preserving its three-channel format, enabling direct use of pretrained CNN models designed for standard RGB inputs. For each modality, the datasets were split into an 80:20 ratio for training and testing, respectively. To enhance model robustness, data augmentation techniques, including horizontal flips, vertical flips, and mirroring, were applied to the training set.

To train the CNN models, cross-entropy loss was used as the loss function, with AdamW as the optimizer. To mitigate overfitting, a dropout of 0.5 and weight regularization were applied. Hyperparameter tuning for each CNN model was performed using a grid search, evaluating different combinations of batch sizes (16 and 32), number of epochs (10, 15, and 20), and learning rates (1 × 10^−5^, 1 × 10^−4^, and 1 × 10^−3^). A one-cycle cosine decay schedule was applied to dynamically adjust learning rates, reducing them to 0.001 times their initial value at the end of training. Model performance was assessed using 5-fold cross-validation on the training set, with the optimal configuration determined based on the average F1_score. The best hyperparameter combination for each model was then used to train and test the model 10 times. The final rankings were based on the average accuracy across all runs.

### 2.7 QTL analysis for architectural traits

Phenotypic data were obtained using both conventional and HTP approaches. Conventional phenotyping classified GH into six categories and MP into four to capture finer-scale trait variation. In contrast, our HTP-based trait classification was simplified by reducing both traits to their two main categories: Bunch or Spreading for GH and Apparent or Not Apparent for MP. This simplification optimized trait estimation while preserving as much phenotypic variability as possible, ensuring consistency across populations and enabling a robust comparison of QTL detection between phenotyping methods.

#### 2.7.1 Genotyping and linkage map construction

Genomic DNA was extracted from leaf tissue of all genotypes using the DNeasy Plant Mini Kit (Qiagen, Hilden, DK) following the manufacturer's protocol. DNA quantification was performed using the Quant-iT Picogreen dsDNA assay kit (Thermo Fisher Scientific, Waltham, MA) according to the manufacturer's instructions. The samples were then submitted to Affymetrix for genotyping with the Axiom_Arachis2 48K SNP array [41,42]. SNP calling was conducted using Axiom Analysis Suite (Thermo Fisher Scientific, Waltham, MA), retaining only high-quality SNPs classified as poly-high-resolution for GT and IF populations. For the C1803 population, Khufu was used for SNP calling [43]. Genetic mapping followed the pipeline described by Chu [44]. Data filtering criteria included: 1) selection of polymorphic SNPs between parental genotypes, 2) exclusion of markers exhibiting segregation distortion (1:1), and 3) removal of redundant loci.

The genetic map for the GT population was constructed using 3969 loci across 123 individuals, with ‘a’ corresponding to Tifrunner and ‘b’ to GT-C20. For the IF population, the map was built using 1687 loci for 145 individuals, where ‘a’ represented Florida-07 and ‘b’ corresponded to ICG 1471. For the C1803 population, a total of 1754 loci were used to construct the genetic map with 147 individuals, where ‘a’ represented Florida-07 and ‘b’ represented NC3033. Genetic maps were generated using JoinMap v. 4.1 [45], with linkage groups determined at a minimum LOD score of 3.0. The Kosambi mapping function [46] was applied to convert recombination frequencies into map distances, measured in centiMorgans (cM).

#### 2.7.2 QTL mapping and heritability

MP and GH were evaluated both as raw multi-category data (conventional phenotyping) and as binary traits (0 and 1) for HTP-based phenotyping. QTL mapping was conducted using recombinant inbred lines (RILs) that displayed consistent values across all three replicates, while non-consistent RILs were excluded from the analysis. Broad-sense heritability was estimated using the *lme4* R package [47].

QTL mapping was performed using the *R/qtl* R package [48]. Single QTL scans were conducted using the Expectation-Maximization algorithm [49]. A 95 ​% Bayesian credible interval approach was used to determine confidence intervals for significant markers. The significance of LOD scores was assessed using 1000 permutation tests, with a significance threshold set at *ρ* ​= ​0.05. To investigate potential interactions and linkages between loci, a two-dimensional genome scan was performed using the *scantwo* function. Additionally, Multiple QTL Mapping (MQM) analysis was applied as an extension of the initial interval mapping to refine the results [50]. A ‘Stepwise’ model selection was applied with a maximum of 8 QTL and penalties of 0.05 ​%. QTL interval was defined as 1.5 LOD from the peak LOD score.

### 2.8 Performance evaluation

To assess the performance of our segmentation and trait estimation pipeline, we conducted a series of evaluations focusing on segmentation accuracy, trait estimation reliability, and overall robustness. The primary objectives were to ensure the precision and consistency of the segmentation process and the accuracy of the derived agronomic traits.

#### 2.8.1 SAM-based field identification

To evaluate the robustness of SAM-based field identification, we tested segmentation consistency under varying orthoimage orientations to simulate different geographic alignments of the field. The RGB orthoimage was incrementally rotated in 0.5° steps from 0° (North-oriented) to 180° (South-oriented). At each rotation angle, we applied SAM's auto-mask generator to extract the field mask, its boundary (minimum oriented bounding rectangle), and the center of this bounding rectangle. This analysis allowed us to assess the stability and accuracy of field segmentation across different orientations.

To quantify segmentation stability, we computed the intersection over union (IoU) between the bounding rectangle obtained at each rotation angle and a manually annotated ground truth rectangle encompassing the field's extent. The IoU metric Equation (1) provides a direct measure of overlap between the predicted field boundaries and the ground truth, ensuring robustness against field orientation changes. While IoU has been used in previous studies to assess segmentation accuracy [51], our approach extends this by evaluating segmentation stability under varying field orientations, providing a more comprehensive validation of the robustness of our methodology.

(1) $$IoU=\frac{\text{area ​of ​intersection}}{\text{area ​of ​union}}$$

where the area of intersection corresponds to the number of pixels within the overlapping region between bounding rectangles, and the area of union is the total number of pixels contained in both bounding rectangles. The IoU metric ranges from 0 ​% (no overlap) to 100 ​% (perfect alignment).

#### 2.8.2 Individual plot segmentation

To evaluate the accuracy of individual plot segmentation, we compared the automated segmentation results with manually annotated ground truth data. Using the LabelMe interactive tool [52], we manually annotated one-third of the field plots (approximately 900 single plots). This subset was chosen to ensure a sufficiently large and representative sample while maintaining the feasibility of manual annotation. These annotations served as ground truth to compute the IoU metric, which quantified the overlap between the predicted segmentation masks and the manually defined masks.

Additionally, segmentation performance was assessed using pixel-wise *Specificity* to assess the detection of positive pixels, *Sensitivity*, and Dice coefficient for evaluating overlap accuracy, providing a comprehensive evaluation of the model's ability to correctly identify plot boundaries and distinguish between adjacent plots. These metrics were computed using Equations (2), (3), (4).

(2) $$Specificity=\frac{TN}{TN+FP}\times 100$$

(3) $$Sensitivity=\frac{TP}{TP+FN}\times 100$$

(4) $$Dice=\frac{2\times TP}{2\times TP+FP+FN}$$

where TP (true positives) and TN (true negatives) represent correctly classified pixels for the canopy and background, respectively, while FP (false positives) and FN (false negatives) indicate misclassified pixels. Specificity and Sensitivity metrics range from 0 ​% (no correct classification) to 100 ​% (perfect classification), while the Dice coefficient is expressed as a value ranging from 0 (no overlap) to 1 (perfect overlap).

#### 2.8.3 Canopy height estimation validation

We validated the canopy height estimation by comparing CH values derived from the nDSM with manually collected field measurements using linear regression analysis. To ensure comparability with other studies, performance was assessed using the coefficient of determination (R^2^), root mean squared error (RMSE), normalized root mean squared error (NRMSE), and mean absolute percentage error (MAPE). These metrics were computed using Equations (5), (6), (7), (8). NRMSE was normalized by the observed range of canopy heights in the field (*CH*_max_ ​− ​*CH*_min_) to facilitate interpretation.

(5) $$R^{2}=1-\frac{\sum_{i=1}^{N}{\left(y_{i}-{\hat{y}}_{i}\right)}^{2}}{\sum_{i=1}^{N}{\left(y_{i}-\bar{y}\right)}^{2}}$$

(6) $$RMSE=\sqrt{\frac{1}{N}\times \overset{N}{\underset{i=1}{\sum }}{\left(y_{i}-{\hat{y}}_{i}\right)}^{2}}$$

(7) $$NRMSE\;\left(\%\right)=\frac{\sqrt{\frac{1}{N}\times \sum_{i=1}^{N}{\left(y_{i}-{\hat{y}}_{i}\right)}^{2}}}{y_{\max}-y_{\min}}\times 100$$

(8) $$MAPE\;\left(\%\right)=\frac{1}{N}\times \overset{N}{\underset{i=1}{\sum }}|\frac{y_{i}-{\hat{y}}_{i}}{y_{i}}|\times 100$$

where *N* is the total number of data points used for linear regression analysis (*N* ​= ​50), *y*_i_ represents the actual CH value for the *i*^*th*^ ground truth plot, $\hat{y}$_i_ is the estimated CH for the *i*^*th*^ plot, and $\bar{y}$ denotes the mean CH value.

#### 2.8.4 CNN-based architectural trait estimation evaluation

To evaluate the generalization capabilities of the trained models for MP and GH classification, we computed average accuracy and recall for each class using Equations (9), (10). These metrics quantified the models’ ability to classify morphological traits accurately and to correctly identify samples from each phenotype class.

(9) $$\text{Accuracy}=\frac{T_{0}+T_{1}}{N}\times 100$$

(10) $${\text{Recall}}_{c}=\frac{T_{c}}{T_{c}+F_{c}}\times 100\;\text{for ​}c\in \left\{0,1\right\}$$

where *T*_0_ and *T*_1_ denote the number of correctly classified samples for class 0 and class 1, respectively; *F*_0_ and *F*_1_ denote the number of samples belonging to class 0 and class 1 that were misclassified as the other class; and *N* is the total number of samples evaluated. For GH, class 0 corresponds to Bunch and class 1 to Spreading; for MP, class 0 corresponds to Apparent and class 1 to Not Apparent. Accuracy and recall values range from 0 ​% (poor performance) to 100 ​% (perfect classification).

## 3 Results

### 3.1 Fully automated workflow for aerial orthomosaic processing

#### 3.1.1 Field identification from orthomosaic using SAM auto-mask generation

**Fig. 5:**
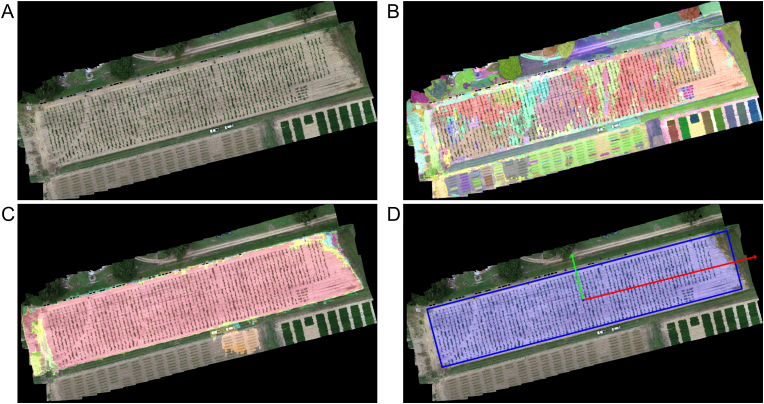
Field masks from SAM auto-mask generator. (A) Orthoimage of the field. (B) Full set of masks automatically generated by SAM. (C) Filtered masks centered in the experimental field. (D) Best field mask with minimum area rectangle enclosing the experimental field. Different colors indicate different segmented image masks. Red and green arrows indicate the orientation of the first and second principal components.

Our approach, based on SAM's auto-segmentation mode, accurately identified the boundaries of the experimental field without manual intervention (Fig. 5). The initial segmentation (Fig. 5B) produced 3072 masks, capturing diverse regions, including the crop field, pathways, and surrounding vegetation. Each sampling point generated three segmentation masks, resulting in some degree of oversegmentation, where certain areas were represented by multiple masks. Post-processing refined the selection by filtering out peripheral and non-crop masks (Fig. 5C). The final selected mask, determined using SAM's stability score and predicted IoU metrics, accurately delineated the experimental field (Fig. 5D).

Field orientation estimates demonstrated robust accuracy across different tested angles (Fig. S1). The maximum absolute error remained below 0.8°, indicating minimal deviation even at extreme orientations. The differences between SAM-derived orientations and the actual field angles followed a normal distribution centered around −0.2°, indicating the consistency of these estimates, with a symmetric distribution and no significant outliers. This level of precision is particularly relevant for subsequent phenotyping steps, as accurate field alignment directly impacts the segmentation and spatial referencing of individual plots. By minimizing orientation errors, our approach ensures that further steps are based on correctly aligned plot structures, reducing potential biases introduced by misalignment.

The accuracy of field boundary detection was further assessed through a comparison with manually annotated field extents, revealing a high degree of agreement (Fig. S2). The IoU values consistently exceeded 0.85, with the majority clustering around 0.95, indicating that the detected boundaries closely matched the actual field limits. The distribution of IoU values followed a negatively skewed normal pattern, with a mean IoU of 0.93 and a skewness of −0.89, indicating a strong tendency toward high overlap, which ensures that the extracted field regions align well with the actual planting area, minimizing errors in subsequent segmentation and phenotypic trait extraction. The slight skewness suggests that while most detections were near-perfect, a small subset exhibited slightly lower accuracy.

#### 3.1.2 Individual plot delineation based on SAM interactive mode

The segmentation of individual plots using SAM interactive mode with multi-point prompts yielded precise delineations, with most plot boundaries correctly identified (Fig. S3). The use of temporary plot centroids enabled the generation of multi-point prompts, guiding the segmentation process effectively. This approach allowed for automated plot-level extraction, significantly reducing the need for manual annotation.

**Fig. 6:**
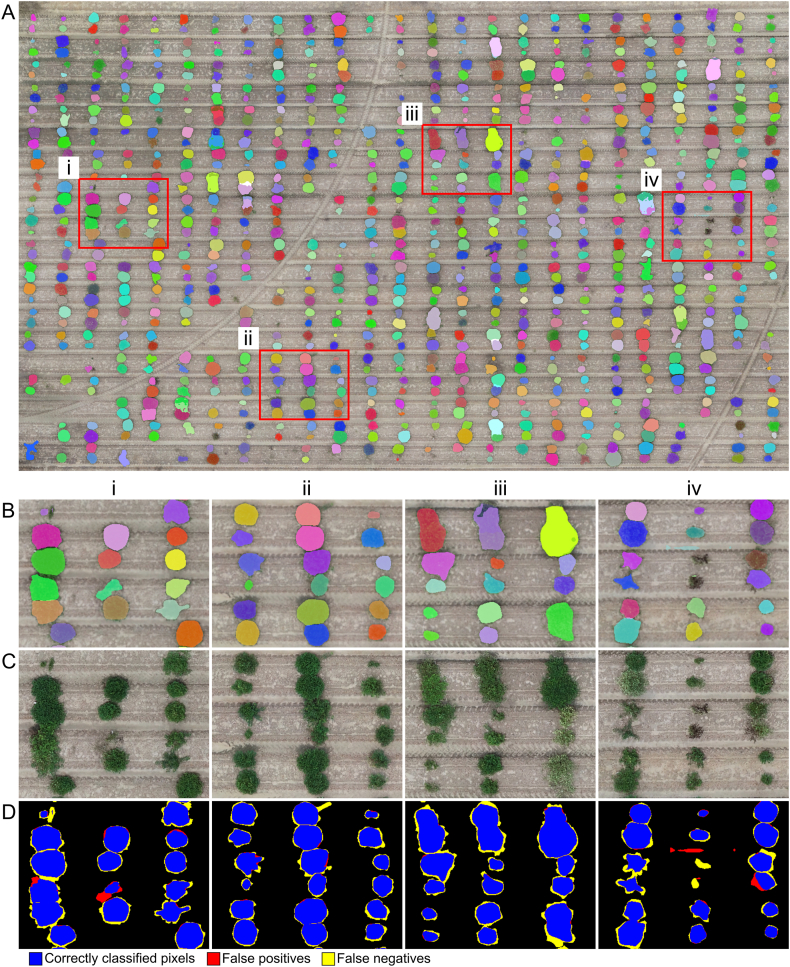
Analysis of plot segmentation errors using SAM. (A) Full-field view showing plot segmentation results. Different colors indicate different segmented plots. Red boxes labeled i–iv indicate regions with prominent segmentation errors. (B) Close-up views of segmentation masks from regions i–iv, highlighting variability in plot shape and overlap. (C) Corresponding raw RGB images for the same regions. (D) Error visualization using color-coded masks: blue indicates correctly segmented plot pixels, red represents false positives (pixels incorrectly included in plots), and yellow shows false negatives (missed plot pixels).

A detailed evaluation of the segmentation masks revealed several challenges, particularly in canopy delineation and detection of small or irregular plots (Fig. 6). Our SAM-based approach successfully segmented overlapping plots (Fig. 6, insets i and ii). However, some masks were duplicated at the two-row plot level (Fig. 6, inset iii), likely due to similar texture patterns between adjacent plots. Although SAM occasionally produced over- or under-segmented masks within individual plots, particularly in slender or irregularly shaped plots, these artifacts were confined within the same two-row unit. Since all agronomic traits were estimated at the plot level (i.e., encompassing both rows on the same raised bed), these internal segmentation variations did not affect downstream trait analysis. We confirmed that canopy height, growth habit, and main stem prominence estimates remained consistent across plots, including those with minor segmentation ambiguity.

Notably, false negative errors were observed where portions of the canopy were missed, especially along plot boundaries. These omissions likely result from the reduced resolution of input images, which limits the model's ability to capture fine details—particularly in regions with complex textures and shading. In some instances, entire plots were only partially segmented (Fig. 6, insets i and iv), potentially due to variations in plant density or atypical plot geometries.

False positives were also present, with senescent vegetation occasionally misclassified as part of the canopy (Fig. 6, insets i and iv), indicating challenges in distinguishing dead plant material from surrounding textures. Smaller or irregularly shaped plots were particularly susceptible to segmentation errors, with terrain pixels occasionally misclassified as part of the plots (Fig. 6, inset i). These findings highlight the need for further refinement, such as morphological filtering, to improve segmentation accuracy by removing spurious elements based on spatial context. Despite these limitations, the SAM-based method demonstrated considerable potential as a scalable and effective solution for automated plot segmentation.

The performance of SAM for individual crop plot segmentation was quantitatively assessed by comparing the segmentation masks provided by SAM with the manually annotated ground truth masks (Table S3). SAM achieved a specificity of 0.99, indicating high accuracy in identifying non-plot areas and minimizing false positives. The sensitivity score reached 0.87, reflecting the model's effectiveness in capturing most crop plots, although some canopy pixels were missed. The Dice coefficient, at 0.92, demonstrates a strong balance between precision and recall, indicating reliable boundary delineation even in the presence of adjacent or overlapping plots. These results suggest that SAM performs well for automating crop plot segmentation in UAS-based phenotyping workflows, providing reliable results for large-scale field data processing, despite occasional segmentation errors under complex field conditions. Fig. 6D illustrates the visual discrepancies between the predicted and reference masks, highlighting common sources of error.

In terms of runtime, the fully automated workflow, from field identification to trait classification, required approximately 54 ​min to process the entire field on a standard workstation without GPU acceleration, assuming that orthomosaic and DSM inputs were already available. The most time-consuming step was individual plot segmentation using SAM, which accounted for 89 ​% of the total runtime (approximately 48 ​min). DSM normalization, including terrain point sampling and nDSM generation, took about 240 ​s (7 ​%), while field boundary identification required around 200 ​s (4 ​%). The final trait classification steps, including canopy height extraction and CNN-based prediction of GH and MP, were completed in under 60 ​s. For a total of 1350 field plots, segmentation took approximately 2 ​s per plot on average, and CNN-based trait classification required less than 0.1 ​s per plot. Once segmentation masks and shapefiles have been generated, the remainder of the workflow, from DSM normalization to trait classification, can be completed in under 6 ​min, making the pipeline highly efficient for large-scale deployment.

### 3.2 Ground truth summary

CH, GH, and MP were assessed manually at 90 days after planting. CH values ranged from 16 to 40 ​cm, with a mean of 24.8 ​cm and a standard deviation of 6.37 ​cm. The distribution of GH and MP classes across populations is reported in Table S4. Most plants exhibited a spreading growth habit, which was the most frequent class in all populations, followed by Bunch types. Intermediate or mixed classes (Spreading&Bunch and Mixed) were less common, while Prostrate and Erect types were rare. For MP, the majority of plants fell into the Not apparent or Apparent categories, with relatively few in the intermediate class. These distributions indicate that the dataset captures the dominant phenotypes while including less frequent variants, providing a representative ground truth for model evaluation.

### 3.3 Plot-level canopy height estimation

**Fig. 7:**
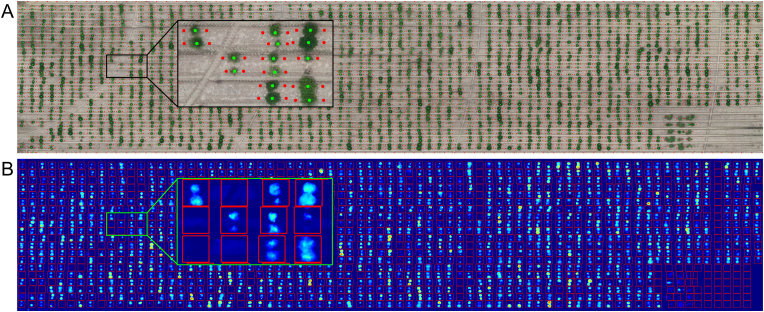
Normalized digital surface model (nDSM) and terrain sampling strategy for height estimation. (A) Visualization of the sampling points used for terrain modeling. Green dots represent provisional plot centroids estimated from SAM-derived segmentation masks, while red dots mark the terrain sampling points. The inset highlights the spatial relationship between vegetation and terrain points. (B) Height-normalized DSM (nDSM), derived by subtracting the ground surface model from the raw DSM. Warmer colors indicate greater vegetation height. The red grid outlines the individual plots used in further phenotypic analysis; the inset shows a zoomed-in view of selected plots.

The automatic terrain sampling method for nDSM reconstruction effectively isolated plant height from underlying terrain variations, ensuring accurate canopy height normalization (Fig. 7). By selecting terrain points adjacent to each plot, the generated DTM corrected for local topographical differences, enabling a precise estimation of crop height across the field. The resulting nDSM (Fig. 7B) provided a consistent height reference for each plot, facilitating a fully automated approach to canopy height extraction.

Using the nDSM, CH estimates were computed by extracting pixel height values within each plot's bounding box and selecting the maximum value as the representative CH measurement. This approach ensured that height estimates were robust to minor variations in canopy structure while maintaining consistency across all plots.

The estimated canopy height exhibited a strong linear relationship with ground-truth measurements (Fig. S4). Our automated method explained approximately 78 ​% of the observed variability, with a low RMSE (just under 3 ​cm) and an NRMSE of 11 ​%, indicating high precision. A MAPE of approximately 10 ​% further confirmed that predicted values closely matched actual canopy heights. Slight underestimations were observed at higher canopy levels, likely due to limitations in UAS image resolution, while minor overestimations at lower heights may stem from terrain model interpolation errors or postprocessing artifacts. Despite these discrepancies, the results demonstrate that our method provides reliable canopy height estimates suitable for HTP applications.

### 3.4 CNN models for growth habit and mainstem prominence estimation

CNN models were only trained for MP and GH, as ground truth data for CH were limited to 50 field-measured samples, which is insufficient to support supervised deep learning. To train robust deep neural networks, particularly for regression tasks where fine-grained continuous predictions are needed, substantially larger datasets are often required to prevent overfitting and ensure broad applicability [53]. Therefore, CH was estimated directly from the nDSM values.

#### 3.4.1 Growth habit

For growth habit estimation, the highest classification accuracy of 88.5 ​% was achieved using the combined RGB and nDSM data with the AlexNet model (Table 1). This result highlights the advantage of integrating structural information from the nDSM with visual features from RGB imagery. Across all tested CNN models, those trained on RGB data alone consistently showed the lowest accuracy, while those trained on nDSM data alone performed better. This indicates the critical role of height and structural information in GH classification.

**Table 1:** Performance of three CNN models across three data modalities in estimating growth habit (GH) and mainstem prominence (MP). Accuracy is reported as the mean and standard deviation (SD) over 10 runs. Recall was computed for each class from average confusion matrices over 10 runs. GH classes: 0 ​= ​Bunch, 1 ​= ​Spreading. MP classes: 0 ​= ​Apparent, 1 ​= ​Not Apparent.

Trait	Data Modality	Model	Accuracy (SD)	Recall_0_	Recall_1_
GH	RGB	AlexNet	0.837 (0.007)	0.821	0.849
		ResNet18	0.844 (0.013)	0.852	0.837
		EfficientNet-B0	0.816 (0.015)	0.842	0.896
	nDSM	AlexNet	0.861 (0.010)	0.859	0.862
		ResNet18	0.864 (0.007)	0.874	0.857
		EfficientNet-B0	0.875 (0.009)	**0.897**	0.881
	Combined	AlexNet	**0.885 (0.007)**	0.881	**0.888**
		ResNet18	0.862 (0.012)	0.877	0.850
		EfficientNet-B0	0.844 (0.014)	0.869	0.825
MP	RGB	AlexNet	0.792 (0.013)	0.713	0.850
		ResNet18	0.789 (0.024)	0.765	0.807
		EfficientNet-B0	0.777 (0.011)	0.716	0.822
	nDSM	AlexNet	**0.834 (0.015)**	**0.781**	0.881
		ResNet18	0.830 (0.017)	0.748	**0.890**
		EfficientNet-B0	0.826 (0.014)	0.768	0.870
	Combined	AlexNet	0.810 (0.011)	0.745	0.857
		ResNet18	0.812 (0.014)	0.761	0.849
		EfficientNet-B0	0.795 (0.017)	0.741	0.833

The combined modality further improved performance for AlexNet and ResNet18, demonstrating the potential of multimodal data integration. AlexNet showed the most consistent improvement, with accuracy increasing by about 2.4 ​% from RGB (83.7 ​%) to nDSM (86.1 ​%) and an additional 2.4 ​% with the combined modality (88.5 ​%), indicating its ability to leverage both visual and structural information effectively. ResNet18 showed more modest improvements, with accuracy increasing from 84.4 ​% (RGB) to 86.4 ​% (nDSM) and to 86.2 ​% (combined), suggesting some limitations in fully integrating multimodal data. EfficientNet-B0 performed best when using nDSM alone, reaching 87.5 ​% accuracy, but its performance declined to 84.4 ​% when trained on the combined modality. This may be due to overfitting or feature redundancy, highlighting a possible need for more training data to fully exploit multimodal inputs in deeper models.

Differences in recall between Bunch and Spreading phenotypes were observed across models and data modalities. The use of nDSM consistently improved recall for the Bunch type, indicating that structural information is particularly effective for identifying more erect growth habits, which may be less visually prominent in RGB images and thus harder to classify accurately using RGB alone. In RGB-only models, recall was often less balanced between phenotypes. For example, EfficientNet-B0 achieved a higher recall for Spreading (0.896) than for Bunch (0.842), suggesting that RGB data may not fully capture the structural traits that characterize the Bunch phenotype. In contrast, combining RGB with nDSM led to more balanced recall. For instance, AlexNet with nDSM showed nearly identical recall values for Bunch and Spreading (0.859 and 0.862), reflecting improved classification balance. The best overall performance was observed with the combined modality using AlexNet, where both phenotypes were classified with high and nearly symmetric recall (0.881 for Bunch and 0.888 for Spreading), reinforcing the advantage of integrating structural and visual features. However, in some models, the addition of RGB led to a slight decline in recall for Spreading (e.g., EfficientNet-B0), possibly due to redundancy or suboptimal fusion of multimodal inputs.

#### 3.4.2 Mainstem prominence

For MP estimation, nDSM data consistently outperformed RGB and combined modalities across all models (Table 1). This results emphasize the importance of structural features in assessing mainstem prominence at the plot level. Unlike GH, where multimodal data integration improved performance in some models, the combined modality provided little to no additional benefit. This indicates that MP is primarily characterized by vertical structural cues, which are well captured in elevation data but not readily detectable in RGB imagery alone.

Across the three CNN models, AlexNet and ResNet18 achieved comparable accuracy levels for each modality, particularly for nDSM, where their performance was nearly identical (83.4 ​% vs. 83.0 ​%), demonstrating their capacity to extract structural patterns critical for this classification task. EfficientNet-B0 also performed best with nDSM, but achieved a slightly lower accuracy of 82.6 ​%, potentially reflecting its greater sensitivity to limited training data. Notably, performance declined for all models when trained on combined inputs, suggesting redundancy or suboptimal fusion of RGB and structural features.

Recall differences between classes were most pronounced in RGB-based models, where the Apparent class was consistently harder to detect. For instance, AlexNet showed a 14 ​% gap in recall between the Apparent and Not Apparent classes (0.713 vs. 0.850), suggesting that RGB imagery alone may not sufficiently capture the structural cues needed to identify visible mainstems—possibly due to occlusion or low contrast with surrounding vegetation. The inclusion of nDSM improved recall for both classes and reduced this gap. With nDSM, AlexNet achieved more balanced recall scores (0.781 for Apparent and 0.881 for Not Apparent). ResNet18 achieved the highest recall for the Not Apparent class (0.890), highlighting its strong performance in identifying plots lacking visible mainstem.

### 3.5 Comparison of QTL mapping results from conventional and HTP-derived phenotypes

Although CH was estimated from UAS-derived data, the limited number of ground-truth measurements (n ​= ​50) restricted its use in the QTL analysis. This small sample size reduces the statistical power required to detect significant associations, increasing the risk of false negatives and limiting confidence in detected QTLs for this trait. Therefore, we focused our analysis on MP and GH traits.

The QTL analysis for MP and GH was conducted across all populations. However, no QTLs were identified in the C1803 and IF populations using data from either phenotyping method. As a result, subsequent analyses focus exclusively on the GT population. A Principal Component Analysis (PCA) of genotypic data from 117 genotypes of the GT population showed that individuals with similar phenotypic expressions also exhibit genetic similarities. The first two principal components (PC1 and PC2) accounted respectively for 8.13 ​% and 6.7 ​% of the total genetic variation, demonstrating significant genetic diversity.

For MP, individuals were grouped into Apparent (65.7 ​%) and Not Apparent (34.3 ​%) in the HTP dataset, whereas an additional category, Somewhat Apparent (3.1 ​%), was distinguished in the conventional assessment. This reduction to two categories in HTP resulted in a 3 ​% decrease in variability, suggesting that most of the phenotypic signal was preserved despite the simplification. Similarly, for GH, HTP data classified the population into two primary categories, Bunch (49.2 ​%) and Spreading (50.8 ​%), whereas conventional phenotyping identified a more nuanced distribution, including intermediate categories such as Mixed (3 ​%) and Spreading & Bunch (4.4 ​%), alongside the primary categories of Bunch (44.5 ​%) and Spreading (48 ​%). The simplification to two categories in the HTP approach corresponded with 7.4 ​% reduction in phenotypic variability, while still capturing the majority of the observed diversity. Conventional phenotyping retained higher heritability estimates for both traits, with values of 46.23 ​% for MP and 61.58 ​% for GH, compared to 39.49 ​% and 53.38 ​% and, respectively, from HTP-derived phenotypes.

**Fig. 8:**
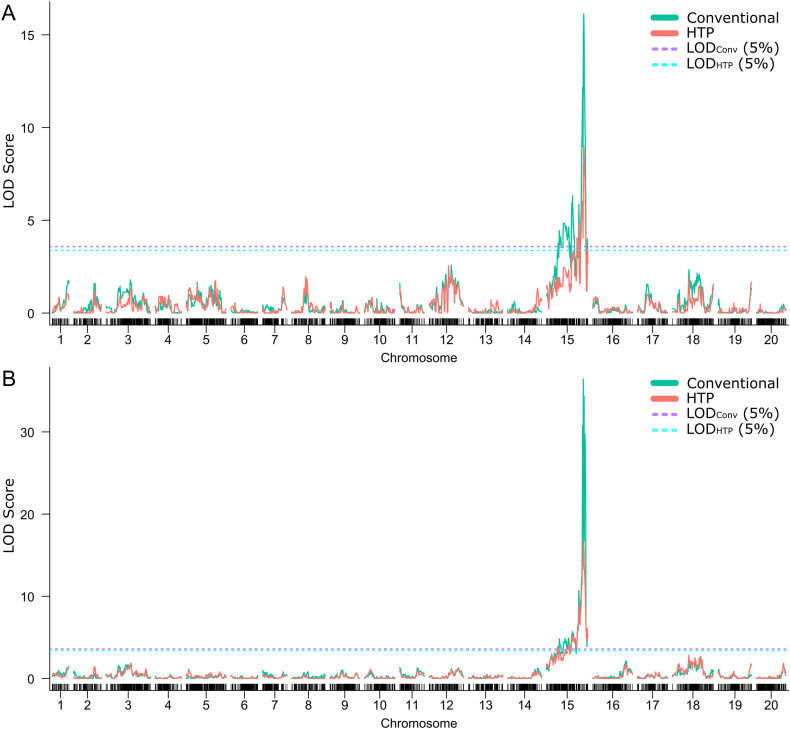
Genome-wide LOD score plots for single QTL mapping across phenotyping method for (A) Mainstem prominence and (B) Growth habit. Significant (*ρ* ​< ​0.05) LOD thresholds based on permutation testing are shown with dotted lines for conventional and high-throughput phenotyping (*LOD*_*Conv*_ and *LOD*_*HTP*_, respectively).

The single QTL analysis revealed consistent evidence of QTLs for both MP and GH on chromosome Arahy.15 across both phenotyping methods (Fig. 8). Both methods identified the same markers, confirming the robustness of the QTLs to phenotyping simplification. Minor differences in QTL parameters were observed, particularly in the lower marker positions when using HTP compared to conventional phenotyping (Fig. S5). The LOD score was slightly lower for HTP, but the same QTLs were identified, demonstrating that the genetic signal remains detectable despite the method's simplification.

**Fig. 9:**
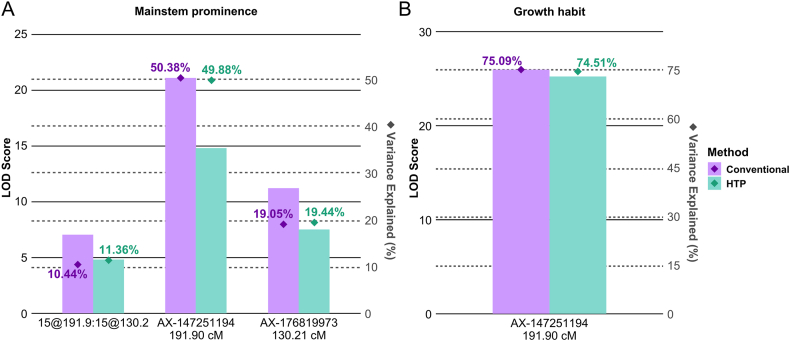
Summary of Multiple QTL Mapping analysis for (A) Mainstem prominence and (B) Growth habit. X-axis indicates identified markers and interaction.

MQM analysis showed that both phenotyping methods identified the same markers and linkage group positions (Fig. 9), indicating consistency in detecting major-effect loci for both traits, irrespective of the method used. For MP (Fig. 9A), both methods detected an interaction between two regions on chromosome B05 (Arahy.15)—one at 191.90 ​cM (marker B05 AX-147251194) and another at 130.21 ​cM (marker B05 AX-176819973). However, notable differences were observed in LOD scores and the proportion of phenotypic variance explained. The QTL linked to B05 AX-147251194 showed a higher LOD score (21.10) and explained a larger percentage of variance (50.38 ​%) with conventional phenotyping, compared to HTP (LOD ​= ​14.81, %Var ​= ​49.88 ​%). In contrast, for B05 AX-176819973, while the LOD score was higher with conventional phenotyping (11.22 vs. 7.52), the variance explained was slightly higher with HTP (19.44 ​% vs. 19.05 ​%). Similarly, for the interaction between loci at 15@191.90 and 15@130.2, HTP explained a slightly larger portion of the variance (11.36 ​%) despite a lower LOD score (4.82), compared to conventional phenotyping (10.44 ​% variance explained, LOD ​= ​7.05). A similar pattern was observed for GH (Fig. 9B), where both methods detected one QTL with comparable LOD scores (25.96 for conventional, 25.23 for HTP) and high variance explained (75.09 ​% for conventional, 74.51 ​% for HTP).

The QTL *qMPB05.1* exhibited a shift in the lower marker interval between the two phenotyping methods for MP, suggesting slight positional variation in detection (Fig. S6). In contrast, the QTLs *qMPB05* and *qGHB05* maintained consistent intervals across both methods, emphasizing their stability. Notably, MP and GH were mapped to the same QTL region, indicating a shared genetic basis.

Regarding parental contributions, for MP-related QTLs (*qMPB05* and *qMPB05.1*), the GT allele was associated with decreased mainstem prominence compared to the Tifrunner allele. In contrast, for GH (qGHB05), the GT allele was linked to a more bunch-type growth habit relative to the Tifrunner allele. Both phenotyping methods detected the same trend for parental contributions.

## 4 Discussion

### 4.1 Effectiveness of SAM for automated orthomosaic processing in breeding fields

The integration of the Segment Anything Model (SAM) into HTP workflows presents a promising path toward automating key steps in field phenotyping. In our study, SAM enabled the automatic identification of field boundaries and individual plots from high-resolution UAS orthoimages. However, while the fine spatial resolution of UAS imagery is beneficial for capturing detailed canopy structure, it also amplifies visual complexity, making small background variations more prominent. As a result, SAM tends to generate excessive masks for irrelevant features, including non-crop elements like pathways, adjacent fields, and shadows. This over-segmentation stems from SAM's lack of semantic understanding, as it cannot distinguish agronomic targets from background elements, requiring robust post-processing strategies [54]. Therefore, effective refinement steps are essential to filter masks and reduce noise.

Boundary delineation from UAS imagery is further complicated by subtle field elements, such as soil texture, weeds, furrows, or canopy gaps, which can mislead segmentation. Compared to satellite imagery, where broader spatial context often simplifies boundary identification [55], the finer detail in UAS data increases the risk of segmentation errors due to the abundance of small, visually similar objects. Additionally, variations in illumination, soil brightness, and canopy heterogeneity can further impact segmentation consistency, demanding adaptive refinement strategies to improve robustness.

Our two-stage refinement approach is conceptually similar to multi-scale segmentation techniques [51], which merge regions based on textural properties. However, our method leverages spatial constraints and geometric filtering. The final mask was selected by ranking candidates based on predicted IoU and stability scores, consistent with metric-based refinement strategies [26]. This approach enhanced the reliability of field boundary extraction, enabling accurate, context-independent delineation.

SAM's interactive mode further allowed automated individual plot segmentation using auto-generated multi-point prompts. This method supports the broader trend of scalable field delineation in phenotyping tools such as Easy MPE [18], GRID [20], and FIELDimageR [19,56]. Our results confirm that SAM could extend these tools' capabilities by eliminating the need for annotated data and manual parameter tuning.

Nonetheless, segmentation errors persist. We observed under-segmentation at plot borders and occasional omission of plots with low canopy density or irregular shape. These limitations are consistent with challenges reported for traditional pixel-based methods [18,57,58] and reflect SAM's sensitivity to planting uniformity and scene complexity. Additionally, visually similar terrain elements led to false positives, particularly when vegetation structure was sparse. These findings suggest future work should explore integration of spatial regularization and morphological refinement to improve segmentation robustness.

While our pipeline demonstrated strong performance in a peanut breeding trial characterized by heterogeneous canopy architecture, further evaluation is necessary to assess its applicability under more complex field conditions. For example, heavy weed pressure can obscure alleyways, complicating the separation of adjacent plots and hindering centroid detection during row segmentation, as noted in prior plot segmentation studies [18,20]. Similarly, in crops prone to lodging, overlapping canopies across neighboring plots may cause the SAM model to generate merged or ambiguous segmentation masks, thereby reducing plot-level precision. Additionally, crops with long, stringy, or trailing plant parts may pose challenges for SAM, as such structures tend to produce irregular and entangled shapes that are difficult to distinguish. These challenges reinforce the need to develop complementary filtering techniques or structure-aware refinements that leverage height and spatial information to improve delineation of individual plots. Expanding this workflow to address such complexities is a critical step toward broader adoption across diverse crop types and planting scenarios.

Several aspects of the proposed workflow also represent meaningful innovations over prior plot extraction tools such as Easy MPE [18] and GRID [20]. First, our method demonstrates greater adaptability to complex field heterogeneity. By combining SAM's zero-shot segmentation capabilities with automated point prompt generation, our pipeline accommodates irregular planting, missing plots, and localized terrain variability without manual parameter tuning or assumptions of field uniformity. Second, the use of SAM's vision transformer architecture enables generalized, prompt-based segmentation without the need for annotated datasets or retraining, making it more scalable across different crops and field conditions than supervised semantic segmentation networks. Third, we integrated localized DTM sampling adjacent to each plot mask, which enhances CH estimation accuracy by reducing the influence of elevation artifacts (e.g., wheel furrows or pivot tracks), a limitation of global terrain models often used in previous pipelines. Finally, our study presents a fully automated pipeline that not only extracts individual plots but also estimates relevant phenotypic traits (GH and MP) using CNN models, thereby offering a comprehensive solution for UAS-based high-throughput phenotyping in breeding programs.

Incorporating SAM into field phenotyping pipelines offers significant benefits. It provides a scalable alternative to manual segmentation, substantially reducing labor and enabling consistent data extraction across trials. Continued development of context-aware post-processing and prompt selection strategies will be essential to maximize SAM's potential in complex agricultural settings.

### 4.2 Precision of automated canopy height estimation

Automating CH estimation is critical for reducing the labor and time demands of manual measurements, thereby improving the scalability of HTP in breeding programs. Our results showed a strong correlation between estimated and measured CH values, demonstrating the effectiveness of the proposed approach. Nonetheless, a slight underestimation of taller plants was observed, likely due to the inherent resolution limits of UAS-based RGB photogrammetry and the challenges of capturing the highest points of dense canopies from a nadir perspective. Minor overestimation of shorter plants was also observed, likely reflecting limitations in terrain modeling and image resolution near the ground, where even small elevation errors can distort DTM [59]. These trends align with earlier findings from peanut canopy studies using UAS-derived data [9]. However, the practical impact of these limitations may be minimal in breeding programs focused on compact canopy architectures, which are commonly favored in peanut breeding for mechanization [60].

Reliable CH estimation depends on accurate DTM reconstruction, especially in heterogeneous fields such as those with raised-bed systems. Traditional UAS workflows often use a single global DTM for the entire field, which may be inadequate for fields with substantial terrain variation between plots. In contrast, our approach employs local terrain sampling adjacent to each SAM-derived segmentation mask, reducing the influence of large-scale elevation artifacts (e.g., wheel furrows) by focusing on localized terrain around each plot. This improves terrain estimation accuracy while eliminating the need for manual terrain point selection. Although minor inconsistencies may arise from natural topographic features near plot edges, such as small soil mounds or pivot tracks, other factors like missing plants or shadows are likely to have limited impact, since sampling is constrained to localized bare-soil regions positioned slightly away from canopy edges. More importantly, by using terrain points local to each plot, potential errors from uneven terrain affect only immediately adjacent plots rather than propagating across the entire field. This contrasts with global DTM approaches, where a single elevation anomaly can introduce errors impacting canopy height estimates for many plots. Automated DTM generation methods, such as terrain classification algorithms [61] and histogram thresholding [62], have been proposed but may struggle in raised-bed systems due to their non-uniform elevation patterns [9]. Similarly, pre- or post-planting UAS flights have been used to estimate terrain [63], but these may not capture within-season elevation changes accurately, limiting their reliability.

### 4.3 CNN-based architectural traits estimation

DL methods, particularly CNNs, have gained significant traction in HTP in recent years [38,64]. Their capacity to extract hierarchical features from image data makes them well-suited for detecting subtle variations in plant architecture. However, the use of CNNs in peanut phenotyping remains limited [30], and to our knowledge, this study represents one of the first applications of CNN-based models to classify two important architectural traits, such as MP and GH, from UAS-derived imagery in peanut breeding.

While ML holds great promise in plant phenotyping, its adoption is often constrained by limited training data and the high cost of manual annotation [64]. Rather than attempting to directly overcome these constraints, we focused on improving model performance by enriching image inputs with structural information. Specifically, we integrated nDSM data into the RGB channels, enhancing the input without increasing the number of channels. This allowed us to use standard pretrained CNN models. Notably, models trained on nDSM data outperformed those using only RGB inputs for both GH and MP classification, demonstrating the value of structural cues in improving classification accuracy, even when data is limited.

Among the three tested models—AlexNet, ResNet18, and EfficientNet-B0—AlexNet delivered the highest GH classification accuracy when using RGB ​+ ​nDSM inputs. This performance supports prior work showing that simpler models can be more effective for specific tasks [65]. In contrast, MP classification was best supported by nDSM alone, with RGB ​+ ​nDSM fusion offering no additional benefit or, in some cases, reducing accuracy. This outcome suggests that RGB data may not provide useful complementary information for MP and could introduce noise. Additionally, the lower performance of EfficientNet-B0 with combined data inputs highlights a broader limitation of deeper models, which may require larger data sets or more sophisticated data fusion strategies, such as feature-level integration or attention-based mechanisms, to fully capitalize on multimodal inputs. Our findings, based on fine-tuning pretrained models with relatively shallow architectures, provide a valuable baseline for future work exploring architectural trait estimation in peanuts using more advanced DL techniques.

In addition to overall accuracy, class-wise recall provided further insights into model behavior across phenotypes. For both GH and MP, models trained on RGB imagery alone exhibited recall imbalances between classes, with lower recall for Bunch and Apparent types, respectively. This suggests that RGB inputs alone may inadequately capture structural characteristics critical for identifying these phenotypes, possibly due to occlusions, low visual contrast, or overlap with surrounding vegetation. The incorporation of nDSM consistently improved recall for both phenotype types and reduced class-wise disparities, particularly for models like AlexNet. For instance, in MP classification, recall for Apparent genotypes improved 7 ​% when using nDSM with AlexNet, while recall for Non-Apparent remained high, indicating a more balanced classification. Similarly, for GH, the use of nDSM led to nearly symmetric recall for Bunch and Spreading types. These trends highlight the importance of structural information for improving sensitivity, particularly for phenotypes that are underrepresented or visually ambiguous in RGB imagery.

### 4.4 QTL analysis and genetic insights

Among the populations analyzed, the GT population exhibited clear genetic loci associated with these traits, which were further cross-validated using ground-based data. Even with a reduction in phenotypic variability when using the HTP method—where GH was classified into only two categories (Spreading and Bunch) and MP into Apparent and Not Apparent—it was still possible to conduct robust QTL analysis. However, while QTLs were successfully detected in the GT population, no significant loci were identified in the other two populations using both phenotyping methods. This may be due to differences in genetic architecture or the presence of multiple small-effect loci, making it more challenging to detect major-effect QTLs. Additionally, environmental influences could have contributed to the lack of significant findings.

The accuracy of QTL mapping depends on multiple factors, including the statistical method's ability to detect and estimate QTL effects, the type and size of the mapping population, phenotyping accuracy, the number and relative contribution of each QTL to the total variance and genetic architecture and heritability of the trait [66]. In our results, HTP- and conventional-based traits exhibited significant variation with moderate to high heritability. Specifically, the heritability for MP was 39.49 ​% (HTP) and 46.23 ​% (conventional), while for GH, it was 53.38 ​% (HTP) and 61.58 ​% (conventional). The slight reduction in heritability in HTP-derived traits is due to simplified phenotypic classification, which may limit the capture of fine-scale variability. Heritability, a key parameter in plant breeding, allows for a comparison of the relative contributions of genetic and environmental factors to trait variation [67]. Moreover, we successfully validated the HTP-based data for identifying MP and GH QTLs.

Using both conventional phenotyping and HTP-based data for MP and GH, we identified three loci on chromosome B05. For MP, a significant additive interaction between *qMPB05* and *qMPB05.1* suggests a combined genetic contribution, potentially enhancing trait expressivity and reinforcing the importance of multi-locus effects in trait expression. Although differences were observed in LOD scores, phenotypic variance explained, and QTL intervals, both methods consistently identified the same markers across linkage groups and positions, demonstrating the robustness of QTL detection regardless of the phenotyping approach. The observed differences between phenotyping methods can be attributed to the reduction in phenotypic resolution in HTP-based data, where traits were simplified into two categories. This categorization may limit the ability to capture subtle phenotypic variations, affecting LOD scores and variance explained. The generally higher LOD scores observed with conventional phenotyping suggest stronger statistical support and potentially less noise in manually collected traits, likely due to more direct and precise measurement. However, in some instances, HTP-derived traits explained a comparable or even slightly greater proportion of the phenotypic variance, indicating their potential to capture complex or subtle trait variation not fully reflected in conventional scoring.

For GH, we found that the parental allele from the *fastigiata* (GT-C20) parent had a significant effect on increasing bunch-type growth habit, while for MP, the effect of increasing mainstem prominence was primarily from the *hypogaea* (Tifrunner) parental allele. Importantly, both HTP-based and ground-based phenotyping consistently detected this trend, demonstrating that HTP-derived GH classification aligns well with traditional field-based assessments. In addition, the QTL identified for GH aligns with previous research, which has also reported this region on chromosome B05 as a significant genetic hotspot for growth habit and growth habit-related traits [3,[68], [69], [70], [71], [72]]. The fact that both methods consistently identified the same QTLs and allele effects highlights that HTP-based phenotyping retains most of the critical genetic information needed for QTL mapping of both MP and GH.

Similar studies in other crops have demonstrated the effectiveness of HTP-based phenotyping in detecting agronomically important QTLs in maize [73] and wheat [74], confirming its reliability in capturing complex traits. Our study further supports these findings by demonstrating that HTP-based phenotyping is an efficient, scalable tool for detecting MP and GH QTLs in peanuts. The promising QTL signals detected using HTP-based traits provide a great opportunity to speed up genetic gain through rapid and low-cost phenotyping. In general, the ability of both methods to consistently identify major QTLs that explain a larger proportion of phenotypic variance highlights the potential of HTP as a scalable and efficient tool for genetic studies, even with reduced phenotypic complexity. The identification of major QTLs for MP and GH through HTP-based phenotyping presents significant advantages for breeding programs. By integrating these QTLs into marker-assisted selection pipelines, breeders can efficiently select for desirable growth habits that optimize canopy structure, enhance yield potential, and improve overall plant architecture [68]. Furthermore, the use of HTP-based phenotyping, along with the development of more reliable and accurate methods, could significantly enhance large breeding trials by facilitating high-throughput screening of diverse germplasm. This would enable the rapid identification of peanut performance under complex environmental conditions [75]. Future research incorporating multi-environment trials and additional HTP-derived traits will further enhance the application of HTP for QTL mapping and genetic selection.

### 4.5 Limitations and future work

Despite the strong performance of the proposed segmentation and trait estimation pipeline, several limitations warrant further investigation. One key challenge lies in the reliance on post-processing heuristics for plot segmentation, which may not generalize well to fields with highly fragmented or irregular layouts. While SAM enabled full automation of plot segmentation, its robustness across diverse field conditions might be limited. Addressing oversegmentation, optimizing prompt selection, and incorporating additional spatial information could improve accuracy and adaptability in more complex agricultural environments. Additionally, our study focused on a single peanut breeding trial; evaluating this pipeline across other field layouts, crop types, and environmental conditions is necessary to assess its broader applicability.

Another important consideration is the applicability of the proposed method in more visually complex or variable field conditions. Although our field trial included a large number of plots and captured substantial heterogeneity, such as missing or poorly germinated plots, irregular planting due to manual sowing, localized plant death, and disturbances caused by irrigation pivot tracks, the pipeline still performed robustly overall. However, certain conditions can challenge segmentation accuracy. For example, dense weed pressure, especially in the alleys or between rows, may obscure the boundaries between columns or rows and hinder the detection of plot centroids during segmentation. Similarly, in other crops prone to lodging, overlapping canopies from adjacent plots could prevent clear boundary delineation, particularly if the vegetation becomes visually entangled. SAM, while effective in diverse segmentation tasks, may struggle with elongated or stringy plant structures that cross plot boundaries, potentially leading to undersegmentation or incorrect mask grouping. Additionally, in cases where soil and canopy colors are visually similar, RGB-based segmentation could be less reliable, particularly in early growth stages or under sparse canopy cover. Future work should explore the performance of this workflow in such challenging conditions and investigate adaptations such as height-aware filtering, vegetation index pre-processing, or prompt refinement strategies to enhance robustness.

A third limitation concerns data imbalance in trait classification. Certain trait categories were underrepresented, potentially affecting model performance. Although we restricted the analysis to the most representative categories, more balanced datasets would improve model generalization. Future work should explore advanced data augmentation techniques or alternative strategies to address class imbalance. Additionally, trait labels were derived from visual field ratings, which are inherently subjective. The use of semi-supervised or self-supervised learning frameworks could reduce reliance on manual annotations while maintaining classification accuracy.

Finally, while embedding structural information into RGB imagery improved GH classification, this strategy showed limited benefit for MP, indicating the need for more advanced multimodal integration techniques. Future work should explore alternative neural network architectures and fusion methods to better leverage complementary information from RGB imagery and structural data, including systematic evaluation of transformer-based backbones.

## 5 Conclusion

This study presents a fully automated pipeline for HTP in peanut breeding, integrating SAM-based segmentation and CNN-based trait estimation from UAS-derived imagery. The workflow successfully automated field boundary detection, plot delineation, and canopy height estimation without manual input, significantly reducing labor and improving scalability. By enabling accurate estimation of key architectural traits such as CH, MP, and GH, this approach addresses a critical gap in the application of HTP to peanut breeding. Additionally, we demonstrated the feasibility of using CNNs for trait classification and showed that HTP-derived measurements can support genetic analysis through QTL mapping with results comparable to those from conventional phenotyping. Although minor differences were observed between phenotyping methods, the HTP-derived measurements successfully identified the same QTLs as conventional phenotyping, confirming the reliability of the automated pipeline for genetic analysis. These findings demonstrate that fully automated image-based HTP methods offer reliable and scalable alternatives to manual assessments, particularly for large populations and traits that are difficult to evaluate directly in the field. The tools developed in this study are valuable for accelerating genetic gains in crop improvement programs.

## Author contributions

J. Rodriguez-Sanchez conceptualized the research idea, designed the methodology, curated the data, and conducted the formal analysis. J. Rodriguez-Sanchez also drafted the original manuscript. R. Martins da Silva performed the QTL analysis and contributed to the interpretation of the results. Y. Chu contributed to the methodology, provided ground truth data and resources, and secured funding. L. Rodriguez contributed to the genetic analysis and interpretation of the results. J. Zhang contributed to the methodology and assisted with data collection and processing. K. Johnsen provided additional resources, supervision, and enhancements to the methodology. P. Ozias-Akins contributed to the methodology, provided resources, and secured funding. C. Li contributed to the conceptualization of the research idea, developed the methodology, secured funding, administered the project, provided resources, and supervised the research. All authors contributed to the review and revision of the manuscript and approved the final version.

## Declaration of generative AI and AI-assisted technologies in the writing process

During the preparation of this work the author(s) used ChatGPT in order to improve the readability and language of the manuscript. After using this tool, the author(s) reviewed and edited the content as needed and take(s) full responsibility for the content of the publication.

## Funding

This work was supported by the Agriculture and Food Research Initiative, project award no. 2022-67013-37365, from the U.S. Department of Agriculture's National Institute of Food and Agriculture.

## Declaration of competing interest

The authors declare that they have no known competing financial interests or personal relationships that could have appeared to influence the work reported in this paper.

## Data Availability

The datasets supporting this study are publicly available on Zenodo [https://doi.org/10.5281/zenodo.17274012]. They include plot-level aerial RGB images and nDSM maps from peanut breeding fields for classification of Growth Habit and Mainstem Prominence. The source code used for data processing and analysis will be made available upon request.
