# Supplementary material for: Aerial imagery and Segment Anything Model for architectural trait phenotyping to support genetic analysis in peanut breeding
Source: Plant Phenomics. 2025 Oct 27;7(4):100126. doi: 10.1016/j.plaphe.2025.100126 (PMC13109298; doi:10.1016/j.plaphe.2025.100126)
Supplement: Multimedia component 1 [file mmc1.pdf]

Aerial Imagery and Segment Anything Model for Architectural Trait Phenotyping to Support Genetic Analysis in Peanut Breeding

Javier Rodriguez-Sanchez, Raissa Martins Da Silva, Ye Chu, Lenin Rodriguez, Jing Zhang, Kyle Johnsen, Peggy Ozias-Akins, Changying Li

Supplementary Materials

Figures

Figure S1. Differences in field orientation estimates using the Segment Anything Model (SAM).

Figure S2. Histogram of intersection over union (IoU) values for SAM-based field boundary identification.

Figure S3. Individual plot masks obtained using SAM with a multi-point prompt.

Figure S4. Linear regression analysis between field-measured canopy height (CH) and canopy height estimated from aerial imagery.

Figure S5. Comparison of genetic marker positions identified on chromosome B05 (Arahy.15) by phenotyping method.

Figure S6. QTL intervals for mainstem prominence (MP) identified on chromosome B05 (Arahy.15) in the GT population.

Tables

Table S1. Visual diagrams and descriptions of the four main growth habit classes in peanut, based on standard trait definitions [1, 2].

Table S2. Parameters used in SAM automatic mode for field boundary segmentation.

Table S3. Quantitative evaluation of SAM segmentation performance compared to manually annotated masks.

Table S4. Visual ratings summary for growth habit (GH) and mainstem prominence (MP).

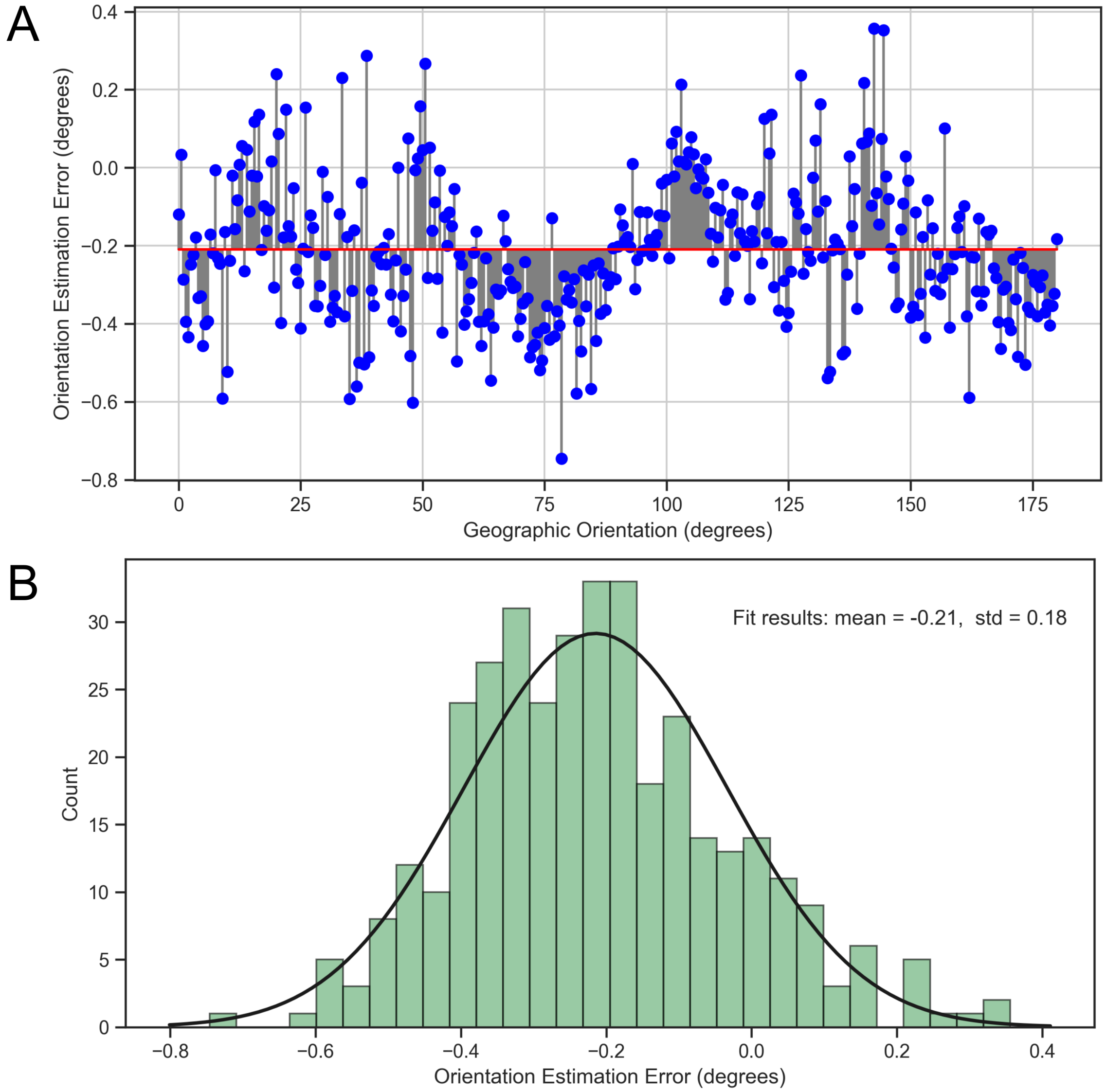

Figure S1: Differences in field orientation estimates using the Segment Anything Model (SAM). (A) Comparison between SAM-based and actual orientation across simulated field directions. (B) Histogram showing angular differences between estimated and actual orientations.

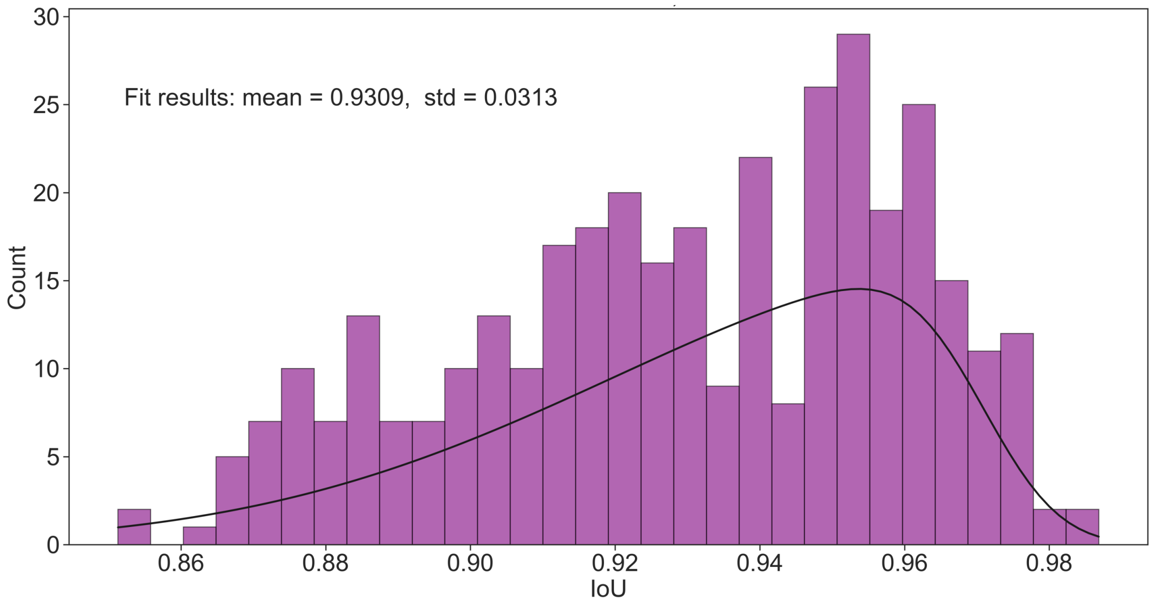

Figure S2: Histogram of intersection over union (IoU) values for SAM-based field boundary identification. The black curve represents the fitted skewed normal distribution (skewness = -0.89).

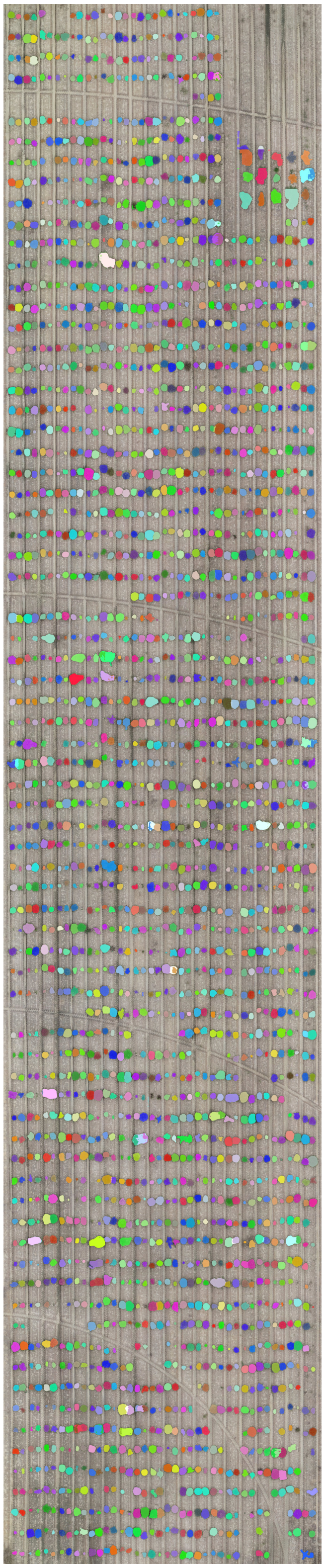

Figure S3: Individual plot masks obtained using SAM with a multi-point prompt. This figure has been rotated 90° for better readability. Different colors represent individual plot masks.

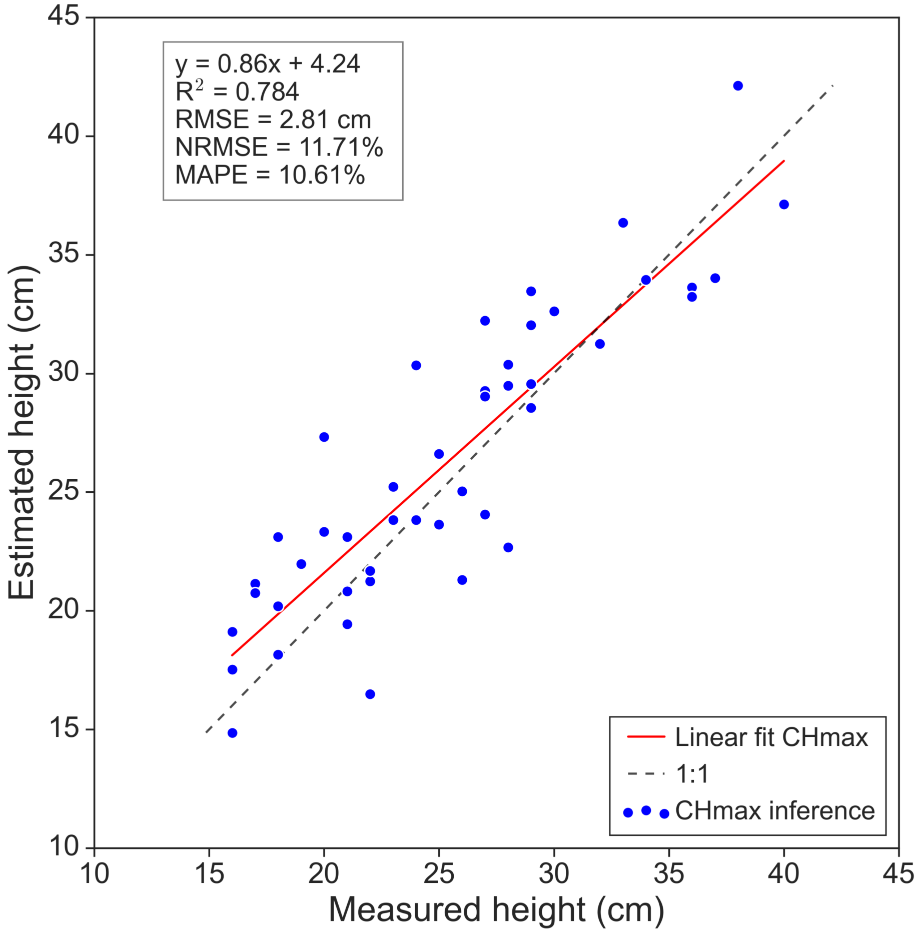

Figure S4: Linear regression analysis between field-measured canopy height (CH) and canopy height estimated from aerial imagery.

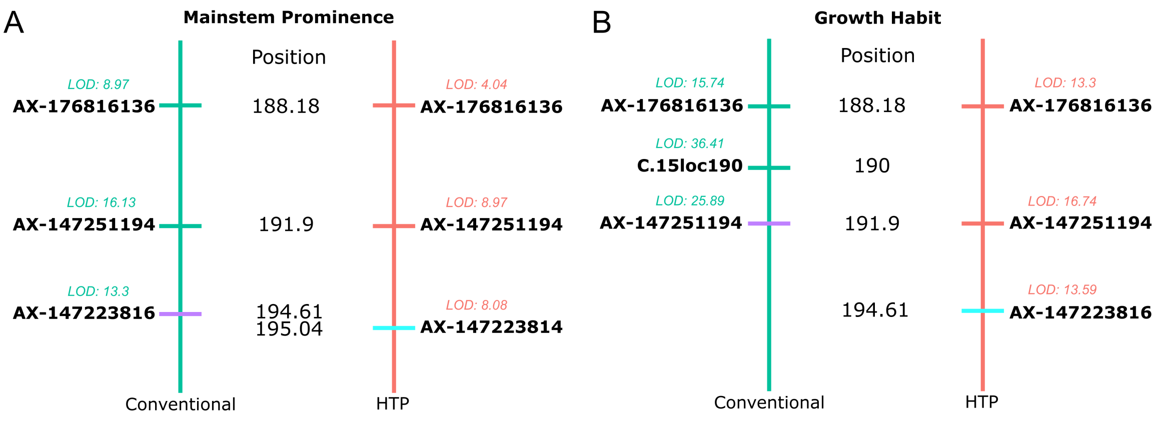

Figure S5: Comparison of genetic marker positions identified on chromosome B05 (Arahy.15) by phenotyping method. Bayesian 95% credible intervals from single-QTL mapping are shown for (A) Mainstem prominence and (B) Growth habit. Green bars indicate QTLs identified using conventional phenotyping, while red bars indicate those identified using high-throughput phenotyping (HTP).

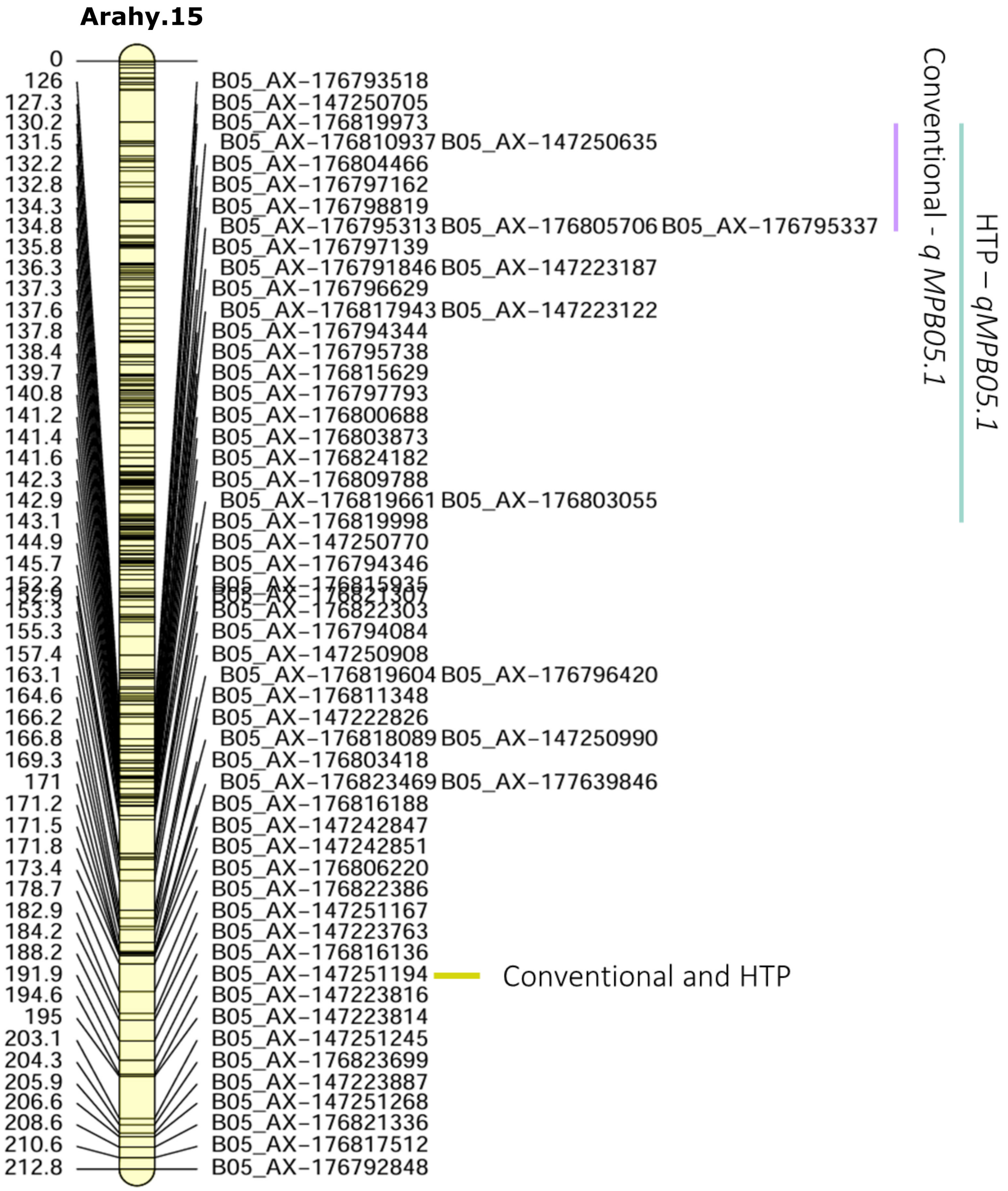

Figure S6: QTL intervals for mainstem prominence (MP) identified on chromosome B05 (Arahy.15) in the GT population. The yellow bar highlights the shared QTL region detected by both conventional and HTP-derived phenotypes.

Table S1: Visual diagrams and descriptions of the four main growth habit classes in peanut, based on standard trait definitions [1, 2].

**Class**	**Score**	**Description**	**Diagram**
Prostrate	1	Branches lie flat on the ground, forming a low, spreading canopy.	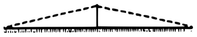
Spreading	2	Branches partially rest on the ground with upward-curved tips.	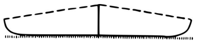
Bunch	4	Branches curve upward near the base, forming a semi-erect canopy.	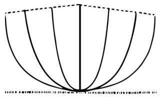
Erect	5	Mostly upright branches with a prominent central stem.	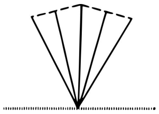

Table S2: Parameters used in SAM automatic mode for field boundary segmentation. IoU (Intersection over Union) defines mask overlap; NMS (Non-Maximum Suppression) thresholds control removal of redundant masks. A threshold of 1 disables suppression.

**Parameter**	**Value**
Grid size (query points)	32×32
Image resize (longest dimension)	1024 pixels
IoU threshold	0
Stability score threshold	0
Box NMS threshold	1
Crop NMS threshold	1
Minimum mask area	10,000 pixels

Table S3: Quantitative evaluation of SAM segmentation performance compared to manually annotated masks.

**Metric**	**Value**	**Interpretation**
Specificity	0.993	Accurate exclusion of non-plot areas (low false positives)
Sensitivity	0.869	Effective plot detection (some missed canopy pixels)
Dice Coefficient	0.916	Strong balance of precision and recall

Table S4: Visual ratings summary for growth habit (GH) and mainstem prominence (MP). Number of samples per class by population.

		Population			
**Trait**	**Class**	**GT**	**C1803**	**IF**	**Total**
GH	Prostrate	0	1	3	4
	Spreading	207	262	216	685
	Spreading&Bunch	19	8	36	63
	Bunch	192	166	170	528
	Erect	0	0	0	0
	Mixed	13	7	0	20
MP	Not apparent	266	255	168	689
	Somewhat apparent	13	13	60	86
	Apparent	145	168	192	550
	Mixed	0	0	0	0

References

1. Pittman RN. United States peanut descriptors. ARS-132. Agricultural Research Service: U. S. Department of Agriculture, 1995.

2. Kayam G, Brand Y, Faigenboim-Doron A, Patil A, Hedvat I, and Hovav R. Fine-Mapping the Branching Habit Trait in Cultivated Peanut by Combining Bulked Segregant Analysis and High-Throughput Sequencing. Frontiers in Plant Science 2017;8. DOI: https://doi.org/10.3389/fpls.2017.00467.
